# Supplementary material for: Dynamics of Gene Expression in Single Root Cells of Arabidopsis thaliana
Source: Plant Cell. 2019 Mar 28;31(5):993–1011. doi: 10.1105/tpc.18.00785 (PMC8516002; doi:10.1105/tpc.18.00785)
Supplement: Supplementary Data [file plcell_v31_5_993_s1.zip › plcell_v31_5_993_s1/TPC2018-RA-00785R1_Supplemental_Data_updated.pdf]

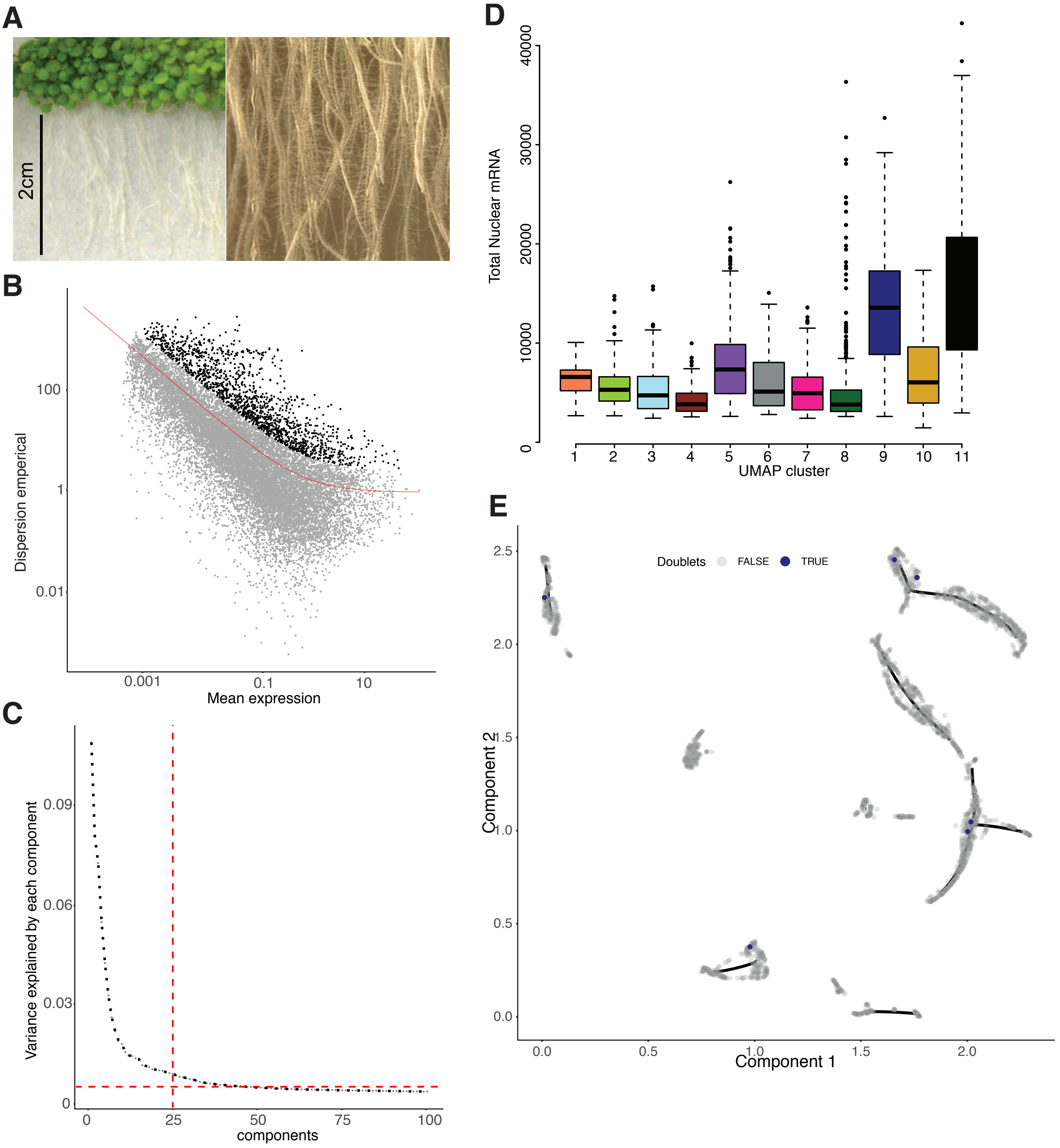

Supplemental Figure 1. General tissue and data features.
Related to Figure 1.
(A) Image of seven-day-old roots used for protoplast preparation. **(B)** Dispersion of gene expression across cells compared to average gene expression. The 1500 ordering genes marked in black had an empirically calculated dispersion at least 2.7 times higher than the dispersion curve fit for the expression of all genes. **(C)** Principal components are plotted against explained variance in gene expression; 25 components were selected. **(D)** Total nuclear mRNA per Louvain component. Louvain components are colored as in Figure 1A.

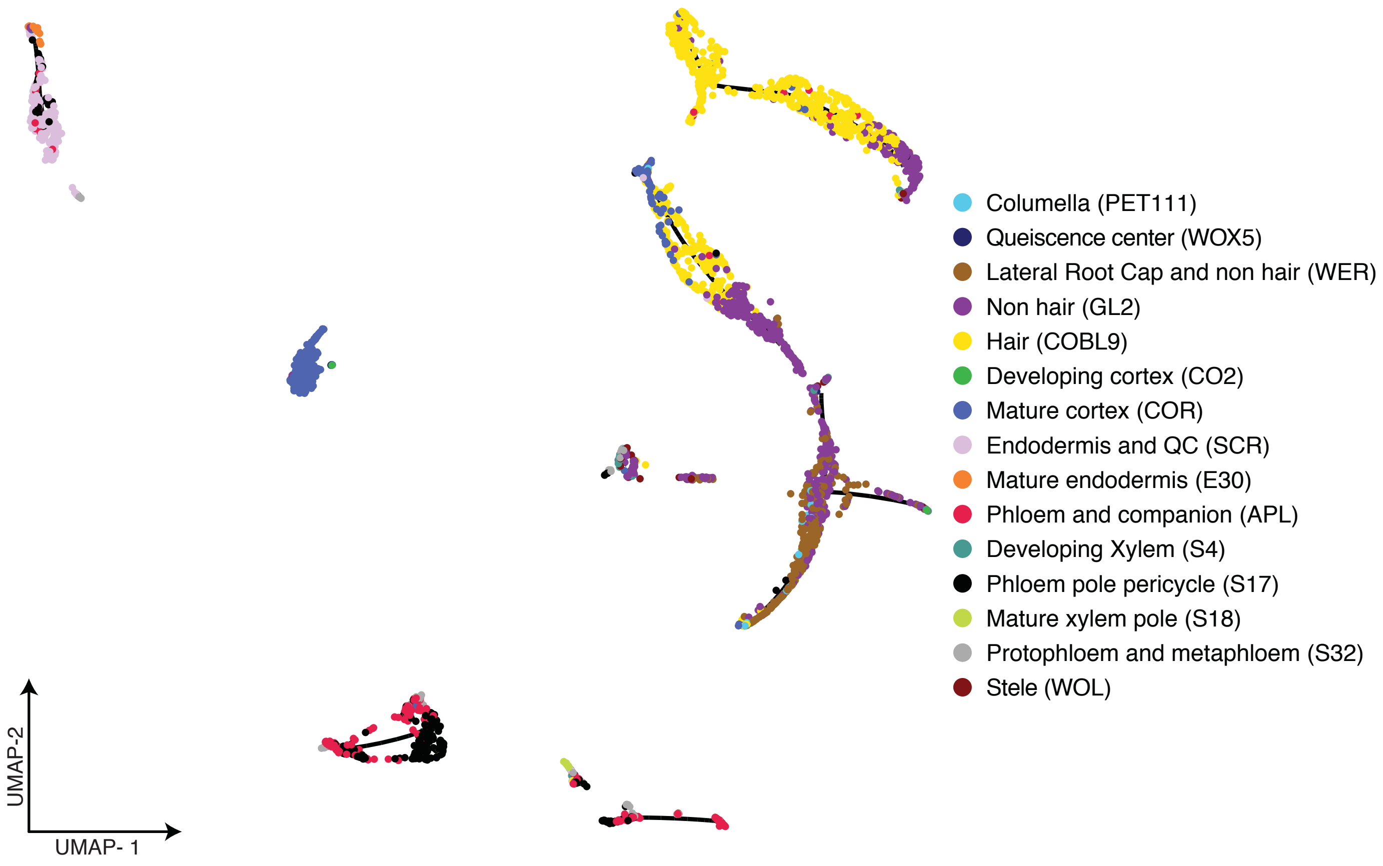

Supplemental Figure 2. Pearson's correlation to sorted bulk RNA-seq samples.
Related to Figure 1.
Correlated expression in each individual cell with previous RNA-seq expression data (Li et al., 2016), assigning cell-type annotation based on the highest Pearson's correlation.

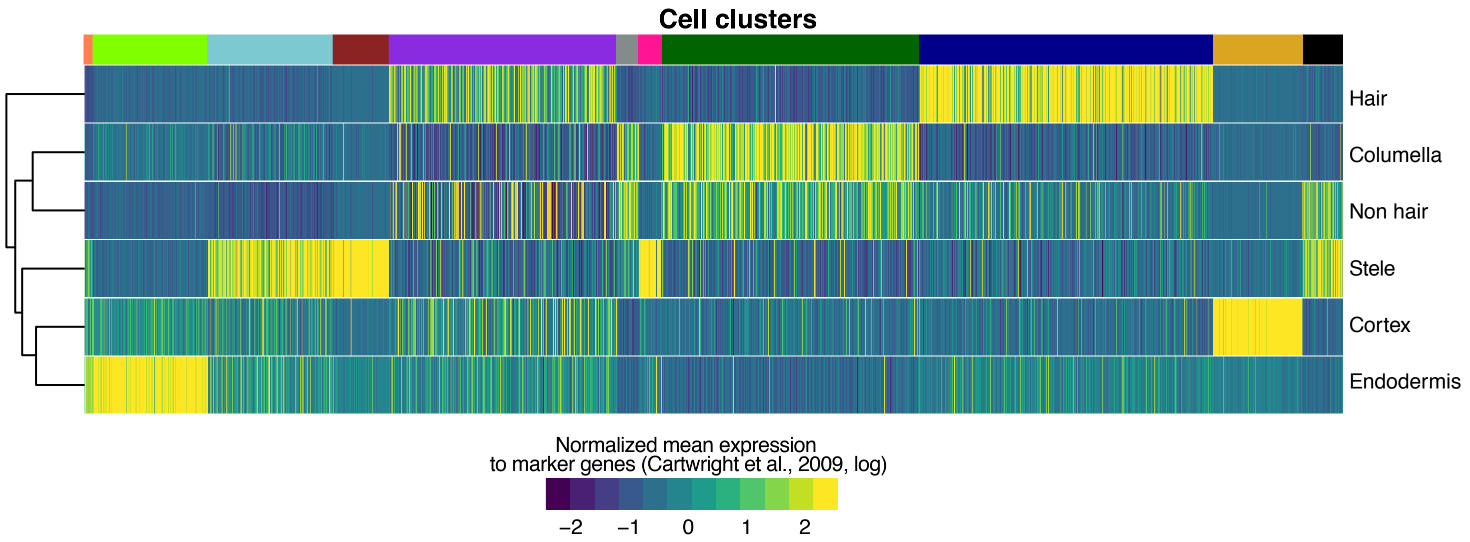

Supplemental Figure 3. Marker gene expression in cell type clusters.
Related to Figure 1.
Known marker gene expression (Brady et al., 2007; Cartwright et al., 2009) was clustered based on expected respective cell types and tissues; mean gene expression is shown for each cell. Cells are clustered by Louvain components across the x-axis.

Supplemental figure 3

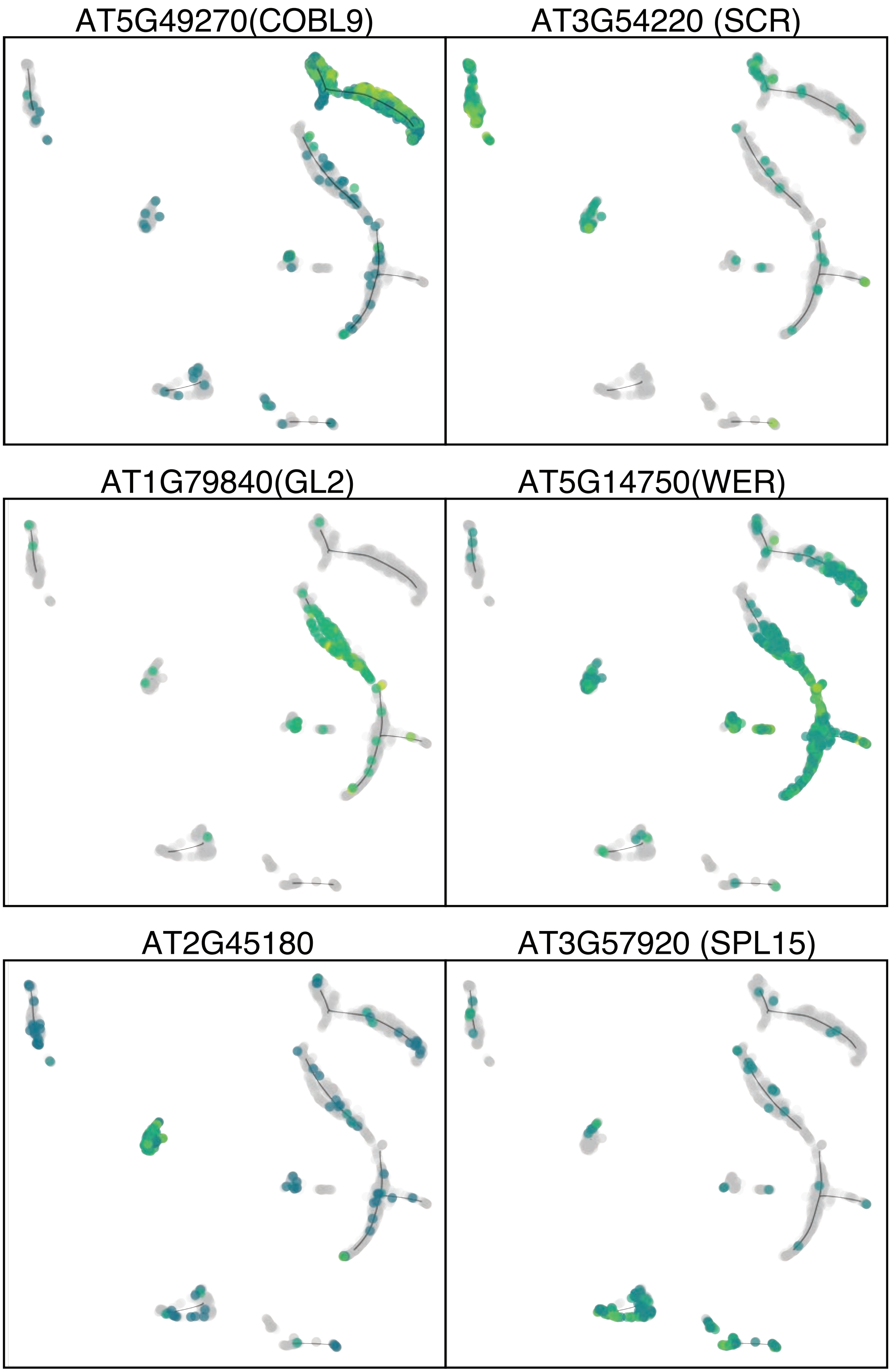

Supplemental Figure 4. Examples of tissue-specific gene expression.
Related to Figure 1.
Expression of several example genes is shown superimposed onto cells across the UMAP clusters; shades of green to yellow indicate expression levels, with lighter color indicating higher expression; cells without detectable expression are in grey. Examples include known and novel genes with tissue-specific expression such as *COBL9*, *SCR*, *GL2*, *WER*, *SPL15* and *AT2G45180*.

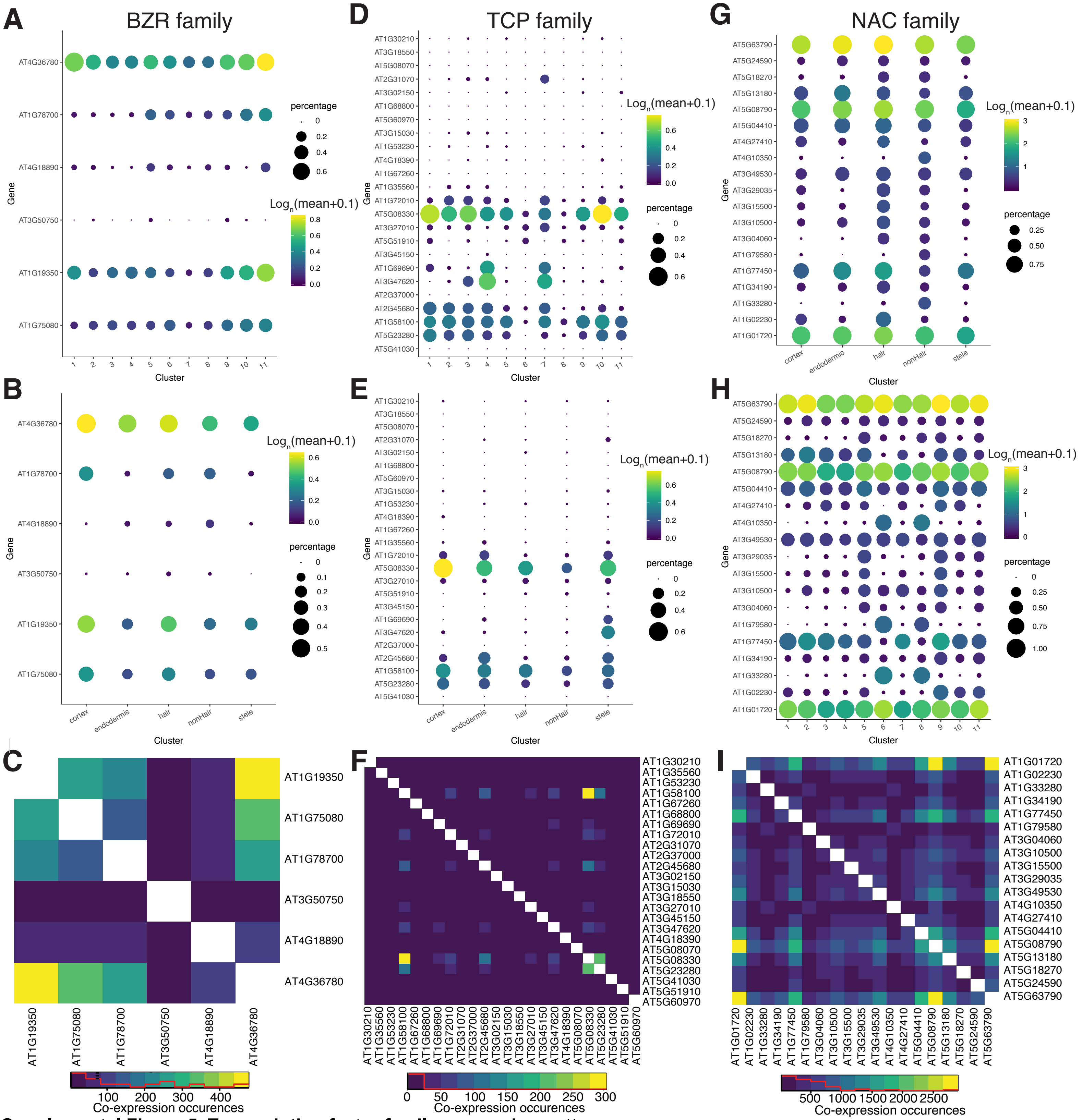

Supplemental Figure 5. Transcription factor family expression patterns.

Related to Figure 2.

(A) Louvain and **(B)** tissue-specific expression for individual members of the BEH/BZR transcription factor family. Circle size indicates proportions of cells expressing a given gene; circle color indicates expression level, with lighter colors corresponding to higher expression. **(C)** *BEH/BZR* gene family members co-expressed in higher numbers of cells with lighter colors. Specific patterns are identified for several genes, e.g. *BES1* (AT1G75080) and *BEH2* (AT4G36780). **(D)** Louvain and **(E)** tissue-specific expression for the TCP transcription factor family. **(F)** TCP gene family members co-expressed in higher numbers of cells with lighter colors. Specific patterns are identified for several TCP members, including for *TCP15* (AT1G69690) and *TCP14* (AT3G47620), two genes primarily expressed in stele. **(G)** Louvain and **(H)** tissue-specific expression for the NAC transcription factor family. **(I)** NAC gene family members co-expressed in higher numbers of cells with lighter colors. Specific patterns are identified for several NAC members, e.g. *ATAF1* (AT1G01720) and *ATAF2* (AT5G08790).

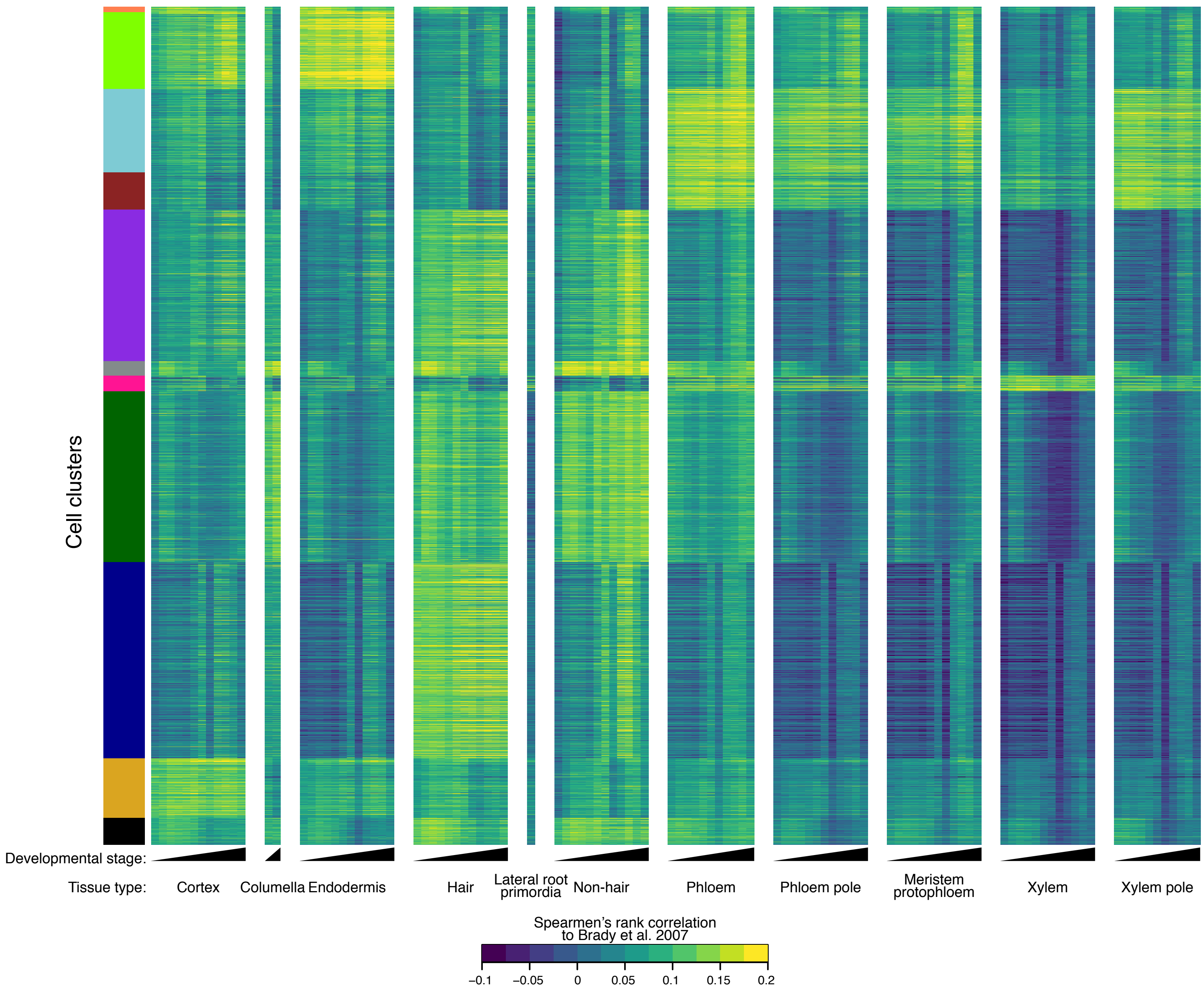

Supplemental Figure 6. Spearman's rank correlation for each cell's development and tissue-type.
Spearman's rank correlation coefficients determined for each tissue and developmental stage (Brady et al., 2007; Cartwright et al., 2009). Development stages are ordered left to right, youngest to oldest. Not all cell and tissue types are represented in every developmental stage; e.g. columella is represented only in stages 1 and 2, lateral root primordia are represented only in stage 12, and phloem is represented from stages 3 to 13. All others are represented from stages 2 to 13.

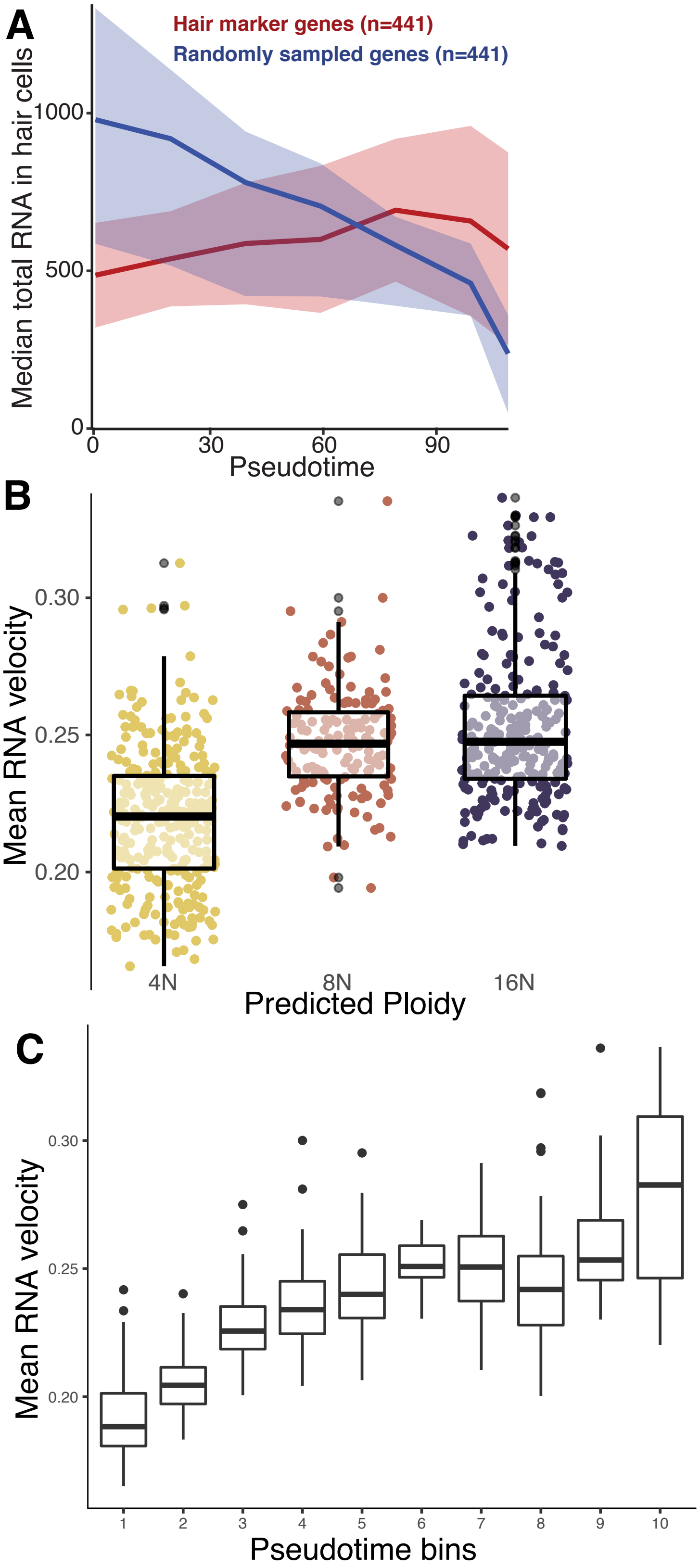

Supplemental Figure 7. Changes in transcription across hair cell development.
Related to Figure 4.
(A) Comparison of median total RNA captured for known hair marker genes (in red, Cartwright et al. 2009) versus a comparable set of random genes (in blue). Median total RNA is plotted; standard deviation is shaded in respective colors. Expression of hair marker genes differed significantly (Permutation test p-value ≈ 1 x 10-4). **(B)** Using published predictions of ploidy in hair cells (Bhosale et al., 2018), and our best rank assignment of developmental time point, we assigned each cell a ploidy and compared RNA velocity across these differing predicted ploidy (Tukey's multiple comparison p-value = 0.0477). **(C)** RNA velocity, an indicator of transcriptional dynamics that relies on the ratio of mature (spliced) to nascent (unspliced) mRNA detected in standard scRNA-seq data (La Manno et al., Nature, 2018), increases over pseudotime in hair cells (Pearson rho = 0.73).

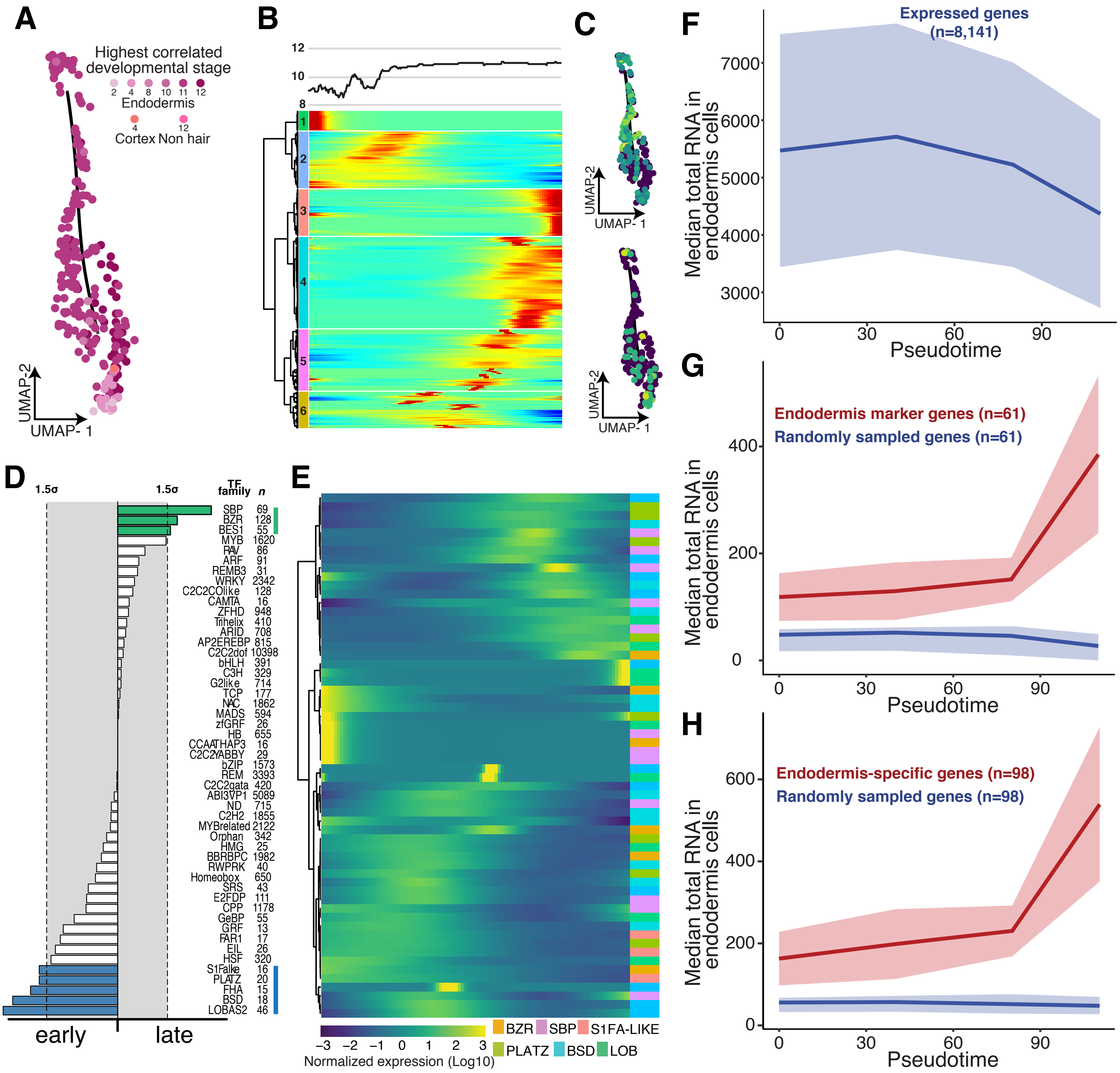
A
Highest correlated developmental stage
2 4 8 10 11 12
Endodermis
4 12
Cortex Non hair
UMAP-2
UMAP- 1
B
12
10
8
1
2
3
4
5
6
C
UMAP-2
UMAP- 1
D
1.5σ
1.5σ
TF family
n
SBP 69
BZR 128
BES1 55
MYB 1620
RAV 86
ARF 91
REMB3 31
WRKY 2342
C2C2COlike 128
CAMTA 16
ZFHD 948
Trihelix 410
ARID 708
AP2EREBP 815
C2C2dof 10398
bHLH 391
C3H 329
G2like 714
TCP 177
NAC 1862
MADS 594
zfGRF 26
HB 655
CCAATHAP3 16
C2C2YABBY 29
bZIP 1573
REM 3393
C2C2gata 420
ABI3VP1 5089
ND 715
C2H2 1855
MYBrelated 2122
Orphan 342
HMG 25
BBRBPC 1982
RWPRK 40
Homeobox 650
SRS 43
E2FDP 111
CPP 1178
GeBP 55
GRF 13
FAR1 17
EIL 26
HSF 320
S1Falike 16
PLATZ 20
FHA 15
BSD 18
LOBAS2 46
early
late
E
−3 −2 −1 0 1 2 3
Normalized expression (Log10)
BZR
SBP
S1FA-LIKE
PLATZ
BSD
LOB
F
Expressed genes (n=8,141)
Median total RNA in endodermis cells
7000
6000
5000
4000
3000
0 30 60 90
Pseudotime
G
Endodermis marker genes (n=61)
Randomly sampled genes (n=61)
400
200
0
H
Endodermis-specific genes (n=98)
Randomly sampled genes (n=98)
600
400
200
0

Supplemental Figure 8. Developmental trajectory of endodermal cells.
(A) UMAP-clustered endodermal cells were assigned a developmental time point based on highest Spearman's rank correlation with bulk expression data of staged tissue (13 developmental stages) (Brady et al., 2007; Cartwright et al., 2009). Cell type and developmental time point are indicated in shades of magenta. **(B)** Cells were ordered in pseudotime; columns represent cells, rows represent expression of the 1500 genes. Rows were grouped based on similarity in gene expression, resulting in 6 clusters (indicated left), with genes in clusters 1 and 2 expressed early in pseudotime and genes in clusters 3 and 4 expressed late. Endodermal cells with the earliest developmental signal (Brady et al., 2007; Cartwright et al., 2009) were designated as the root of the trajectory. The graph above represents the average best-correlation of developmental stage (Brady et al., 2007; Cartwright et al., 2009) in a scrolling window of 20 cells with pseudotime, showing the expected increase in developmental age with increasing pseudotime. **(C)** Examples of an early (AT5G59970) and a late (AT3G59710) expressed endodermis-specific gene; gene expression in each cell is superimposed onto the UMAP cluster, with lighter colors indicating higher gene expression. **(D)** Different transcription factor motifs reside in the 500 bp upstream regions of genes expressed early (clusters 1, 2) compared to genes expressed late (clusters 3, 4). Transcription factor motifs specific to early hair cells are denoted with blue bars, those for late hair cells with green bars; bar length indicates motif frequency. Thresholds on either side (grey box, dotted lines) refer to 1.5 standard deviation above mean motif frequency. **(E)** Expression of individual members of transcription factors families highlighted in D across pseudotime identifies candidate factors driving early or late gene expression. **(F)** Median total RNA captured in cells across endodermis pseudotime. Number of genes included is indicated. **(G)** Comparison of total RNA captured for known endodermis-cell-specific genes (in red, Cartwright et al. 2009) versus a comparable set of random genes (in blue). Median total RNA is plotted; standard deviation is shaded in respective colors. **(H)** Comparison of total RNA for endodermis-cell-specific genes (in red) to a comparable random set of genes (in blue). Number of genes is indicated.

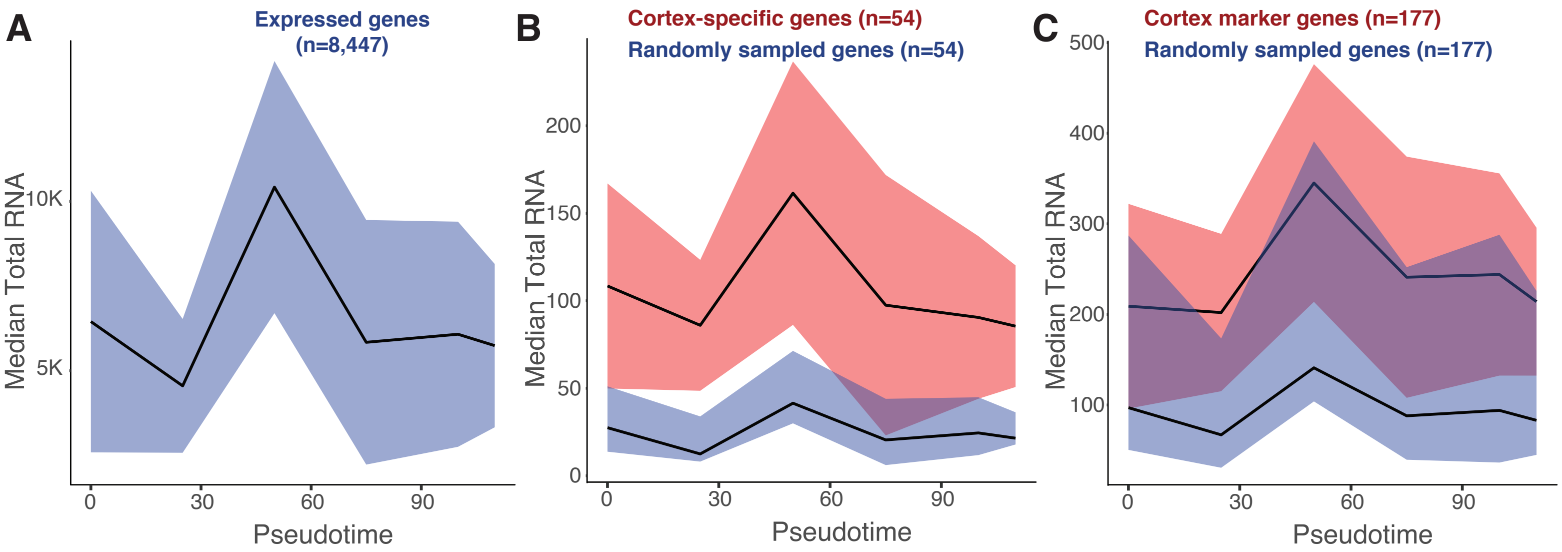

Supplemental Figure 9. Median total RNA in cortex cells across pseudotime.
Related to Figure 5.
(A) Total median RNA captured in cortex cells across pseudotime. Number of genes included is indicated. **(B)** Comparison of median total RNA for cortex-cell-specific genes (in red) to a comparable random set of genes (in blue). Number of genes is indicated. **(C)** Comparison of median total RNA for known cortex genes (in red) to a comparable set of random genes (in blue). Number of genes is indicated.

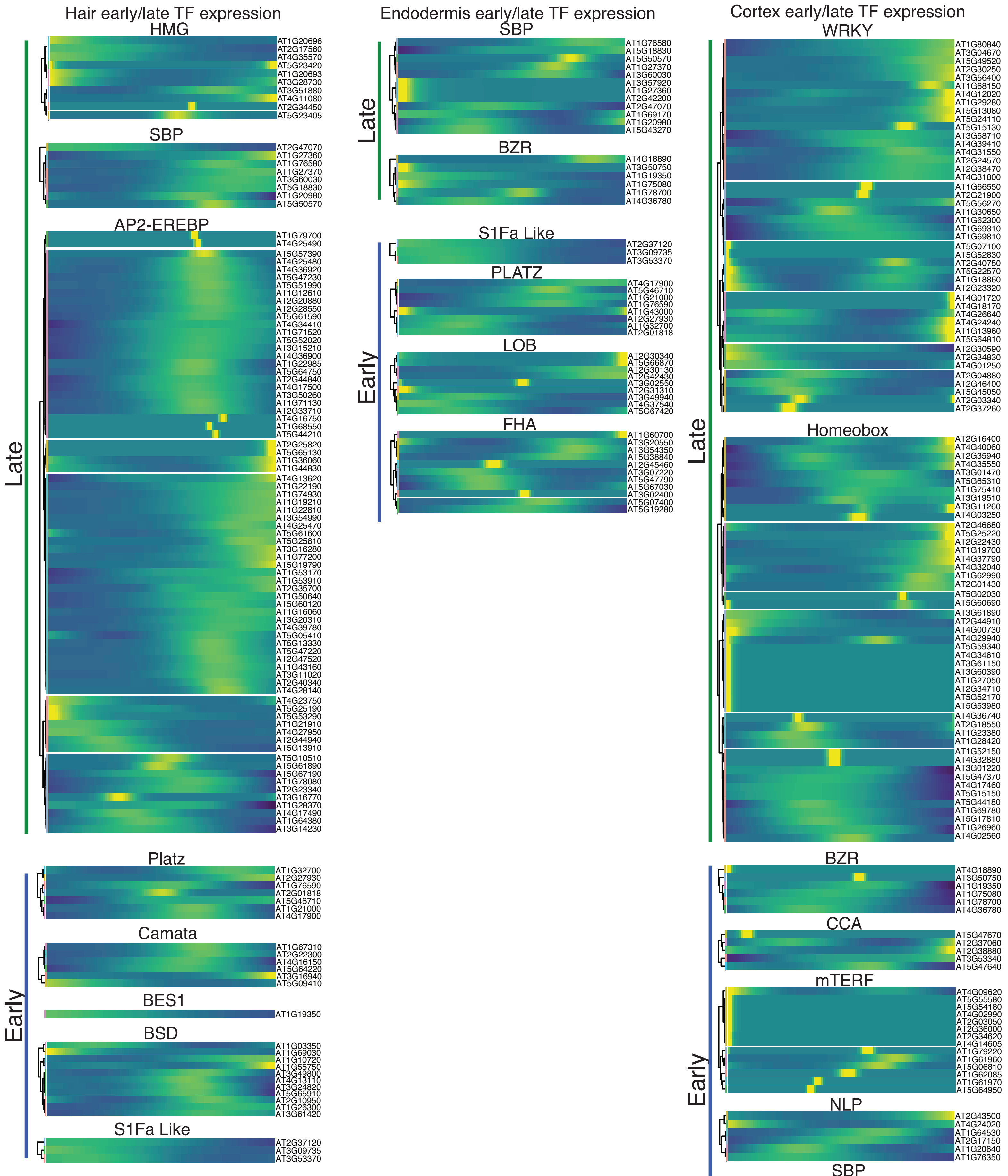

Supplemental Figure 10. Developmental expression of individual transcription factors. Related to Figure 2.
Expression of individual members of transcription factor families with enriched early (indicated with blue bar) or late motifs (indicated in green) for hair, cortex, and endodermis cells. Lighter colors indicate higher expression; x-axis denotes pseudotime progressing from early to late as left to right.

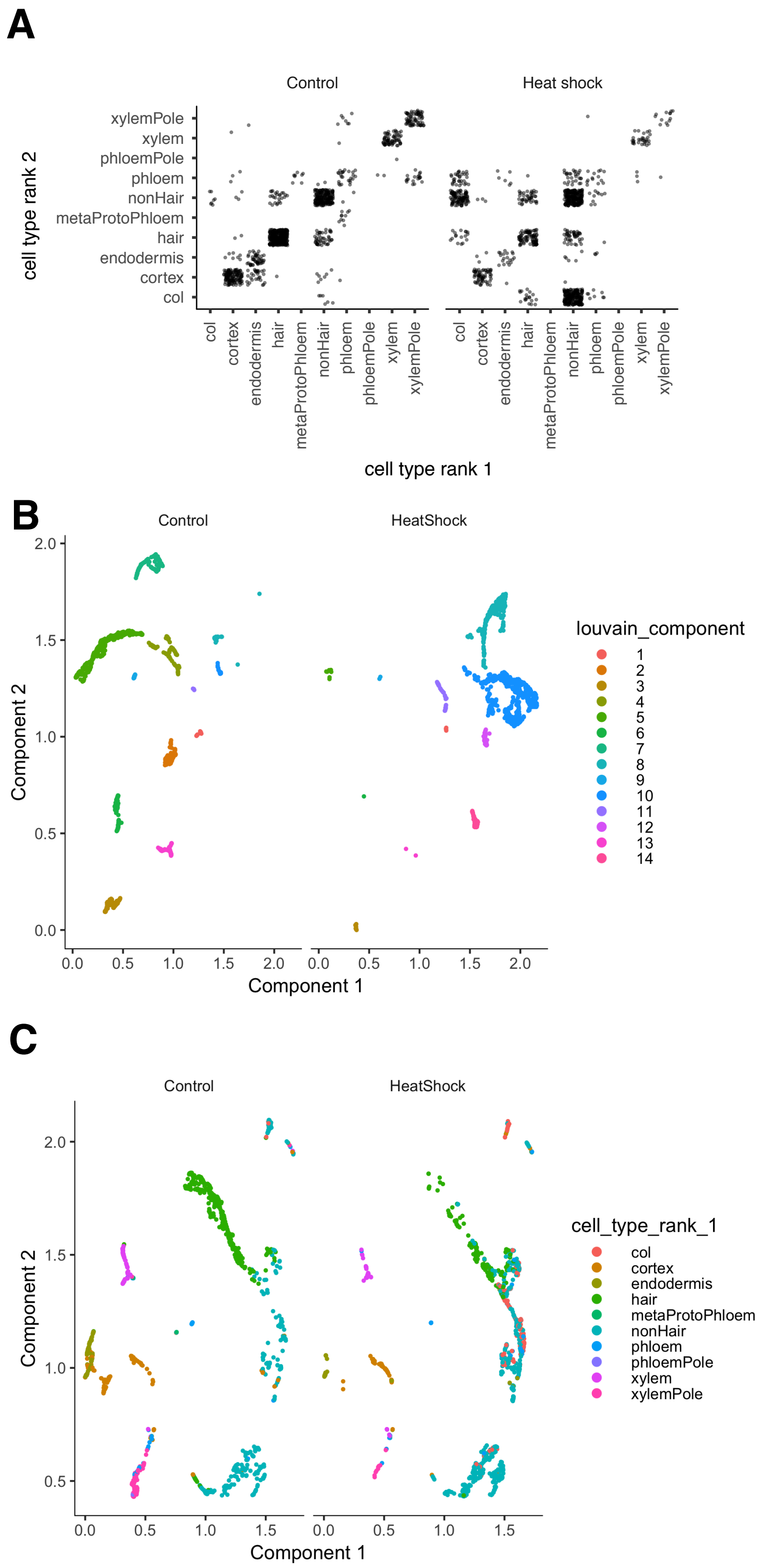

Supplemental Figure 11. Heat-shock clustering and expression profiling.
Related to Figure 7.
(A) Upon heat shock cells are less reliably identified by cell type, as shown by their Spearman's rank correlations across cell types and development. Under control conditions, a cell's second highest correlation is most likely with the same cell type at a different developmental stage (left). Upon heat shock, this relationship between cell ranks is perturbed (right). **(B)** Conventional Monocle clustering and UMAP projection results in cells being primarily clustered by treatment (control or heat shock) rather than cell or tissue type. **(C)** A mutual nearest neighbor approach takes treatment into account to generate comparable clusters; however, some clusters change in size, become ambiguous, or are lost.

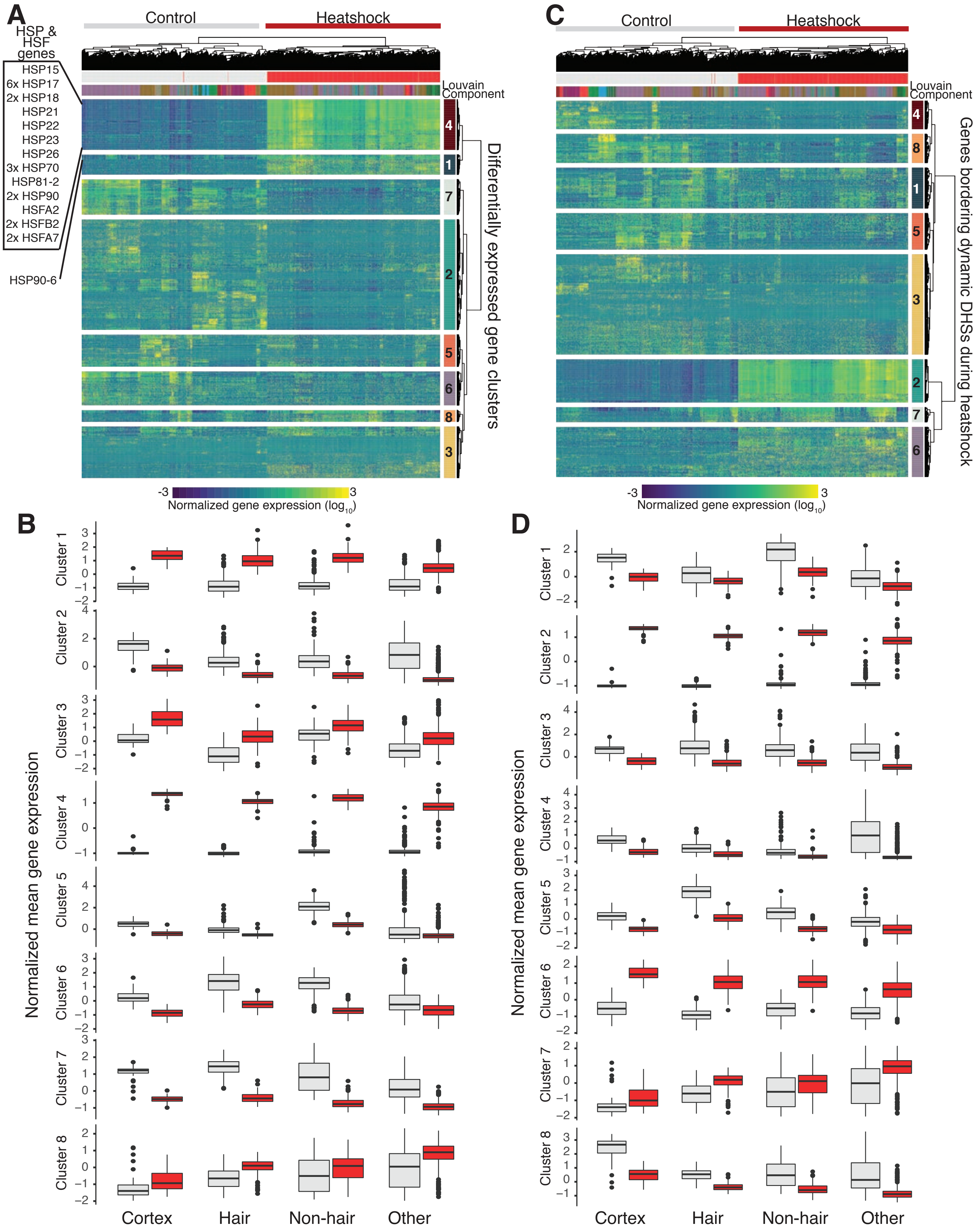
A
Control
Heatshock
HSP & HSF genes
HSP15
6x HSP17
2x HSP18
HSP21
HSP22
HSP23
HSP26
3x HSP70
HSP81-2
2x HSP90
HSFA2
2x HSFB2
2x HSFA7
HSP90-6
Louvain Component
Differentially expressed gene clusters
-3
3
Normalized gene expression (log10)
C
Control
Heatshock
Louvain Component
Genes bordering dynamic DHSs during heatshock
-3
3
Normalized gene expression (log10)
B
Normalized mean gene expression
Cluster 1
Cluster 2
Cluster 3
Cluster 4
Cluster 5
Cluster 6
Cluster 7
Cluster 8
Cortex
Hair
Non-hair
Other
D
Normalized mean gene expression
Cluster 1
Cluster 2
Cluster 3
Cluster 4
Cluster 5
Cluster 6
Cluster 7
Cluster 8
Cortex
Hair
Non-hair
Other

Supplemental Figure 12. Genes known to be associated with the heat shock response show subtle expression differences across clusters.
Related to Figure 7.
(A) Single-cell expression values for genes differentially regulated in response to a similar heat shock in bulk tissue (Alexandre et al., 2018) were clustered based on similarity in gene expression in control versus heat-shocked cells. Clusters are indicated at right. Cluster 4 contains the vast majority of acute heat-shock induced genes, for which chromatin accessibility extends along the gene body upon heat shock (Sullivan et al., 2014). **(B)** Pairwise comparisons of cell and tissue types reveal subtle but highly significant differences in the heat shock response for some clusters. Significance was determined with a generalized linear model. There is no cell or tissue-type specific response for cluster 4, as all cells show the canonical heat stress response typical of genes in this cluster. P-values shown in Supplemental Table 6. **(C)** Single-cell expression values for genes previously identified as residing near chromatin accessible sites that are dynamic in response to heat shock (Sullivan et al., 2014) clustered based on similarity in gene expression in control versus heat-shocked cells. Clusters are indicated at right. Cluster 2 contains the vast majority of acute heat-stress induced genes, for which chromatin accessibility extends along the gene body upon heat shock (Sullivan et al., 2014). **(D)** Pairwise comparisons of cell and tissue types reveal subtle but highly significant differences in the heat-shock response for clusters from genes associated with dynamic chromatin accessibility in A. P-values shown in Supplemental Data Set 6.

Supplemental Table 1. Bulk RNA-seq comparisons to single cell RNA-seq

Bulk GFP Line	Single Cell Group	Pearson Correlation	Spearman's Rank Correlation
**WOL**	WOL expressing cells	0.45	0.66
**WOL**	Stele	0.51	0.68
**WER**	WER expressing cells	0.59	0.75
**WER**	Non-hair	0.54	0.72
**SCR**	SCR expressing cells	0.53	0.72
**SCR**	Endodermis	0.50	0.71
**S4**	Stele	0.37	0.57
**S32**	Stele	0.51	0.69
**S18**	Stele	0.66	0.80
**Pet111**	Non-hair	0.52	0.71
**S17**	Stele	0.66	0.80
**GL2**	GL2 expressing cells	0.58	0.74
**GL2**	Non-hair	0.62	0.78
**COR**	Cortex	0.74	0.86
**E30**	Endodermis	0.26	0.48
**COBL9**	COBL9 expressing cells	0.75	0.87
**COBL9**	Hair	0.74	0.87
**APL**	Stele	0.68	0.82
**CO2**	Cortex	0.3	0.55
**Maturation**	Whole root	0.49	0.70
**Elongation**	Whole root	0.7	0.83
**Meristimatic**	Whole root	0.21	0.42
**Whole root**	Whole root	0.52	0.71

Supplemental Table 2. Number of cells in the control vs. heat shock analysis

Annotation / Louvain Component	Treatment ID	Number of Cells
Columella	Control	7
Columella	Heat Shock	153
Cortex	Control	149
Cortex	Heat Shock	71
Endodermis	Control	77
Endodermis	Heat Shock	26
Hair	Control	371
Hair	Heat Shock	151
MetaProtoPhloem	Control	8
MetaProtoPhloem	Heat Shock	0
NonHair	Control	256
NonHair	Heat Shock	524
Phloem	Control	46
Phloem	Heat Shock	37
PhloemPole	Control	1
PhloemPole	Heat Shock	0
Xylem	Control	70
Xylem	Heat Shock	33
XylemPole	Control	91
XylemPole	Heat Shock	14
Louvain Component 1	Control	24
Louvain Component 1	Heat Shock	5
Louvain Component 2	Control	400
Louvain Component 2	Heat Shock	415
Louvain Component 3	Control	160
Louvain Component 3	Heat Shock	283
Louvain Component 4	Control	97
Louvain Component 4	Heat Shock	18
Louvain Component 5	Control	120
Louvain Component 5	Heat Shock	30
Louvain Component 6	Control	77
Louvain Component 6	Heat Shock	26
Louvain Component 7	Control	60
Louvain Component 7	Heat Shock	2
Louvain Component 8	Control	41
Louvain Component 8	Heat Shock	62
Louvain Component 9	Control	16
Louvain Component 9	Heat Shock	20
Louvain Component 10	Control	43
Louvain Component 10	Heat Shock	132
Louvain Component 11	Control	38
Louvain Component 11	Heat Shock	16
