# Supplementary material for: Dynamics of Gene Expression in Single Root Cells of Arabidopsis thaliana
Source: Plant Cell. 2019 Mar 28;31(5):993–1011. doi: 10.1105/tpc.18.00785 (PMC8516002; doi:10.1105/tpc.18.00785)
Supplement: Supplementary Data [file tpc18.00785.PeerReviewReport.pdf › tpc18.00785.PeerReviewReport.pdf]

Dynamics of Gene Expression in Single Root Cells of *Arabidopsis thaliana*

Ken Jean-Baptiste, José L. McFaline-Figueroa, Cristina M. Alexandre, Michael W. Dorrity, Lauren Saunders, Kerry L. Bubb, Cole Trapnell, Stanley Fields, Christine Queitsch, Josh T. Cuperus

Plant Cell. Advance Publication March 28, 2019; doi: 10.1105/tpc.18.00785

Corresponding author: Josh T. Cuperus cuperusj@uw.edu

Review timeline:

TPC2018-RA-00785	Submission received:	October 31, 2018
	1st Decision:	December 15, 2018 *revision requested*
TPC2018-RA-00785R1	1st Revision received:	February 12, 2019
	2nd Decision:	March 17, 2019 *acceptance pending, sent to science editor*
	Final acceptance:	March 26, 2019
	Advance publication:	March 28, 2019

REPORT: (The report shows the major requests for revision and author responses. Minor comments for revision and miscellaneous correspondence are not included. The original format may not be reflected in this compilation, but the reviewer comments and author responses are not edited, except to correct minor typographical or spelling errors that could be a source of ambiguity.)

TPC2018-RA-00785 1st Editorial decision – *revision requested* December 15, 2018

The editorial board agrees that the work you describe is substantive, falls within the scope of the journal, and may become acceptable for publication pending revision, and potential re-review.

We ask you to pay attention to the following points in preparing your revision.

All expert reviewers found your work interesting and timely. No additional experimental work is requested. However, as you will see from their detailed comments, they all request clarifications to experimental protocols and data analysis as well as better integration with the published literature. In some cases they request additional computational analysis of the data. Particularly reviewer 2 asks for a broader comparison of your data with known expression patterns. Please respond to all of the comments of the reviewers and edit your manuscript thoroughly in light of their suggestions. Your revised manuscript may be sent again to the same reviewers.

--- Reviewer comments:

[Reviewer comments shown below along with author responses]

TPC2018-RA-00785R1 1st Revision received February 12, 2019

Reviewer comments and author responses:

RESPONSE TO EDITOR: We would like to thank the editor and the reviewers for their thoughtful comments and suggestions. In response, we have made extensive changes, conducted several new analyses, and added their results as text and figures to the revised manuscript. Specifically, we have added three new main figures, two new supplemental figures, and extensive modifications to others. We have appreciated the opportunity to clarify our existing analyses and text, which is important given the novelty of the approach and the presented data.

Below, we briefly describe the changes and additional analyses contained in the revised manuscript:

- extended introduction and discussion, including requested citations
- extensive clarification on (i) transcription factor analyses, (ii) the nature of Louvain components, (iii) root developmental stages, (iv) the merit of combining data sets, (v) the overrepresentation of younger cells in our data,

(vi) possible effects of protoplasting on clustering or cell identity, and (vii) and the meaning of RNA velocity

- newly included analysis to identify doublets
- newly included comparison of our data to previously established RNA-seq data of sorted reporter lines to further validate cell type assignments.
- newly included analysis of the stele cluster into individual cell types and identification of novel sub-cluster-specific genes
- newly included analysis of branch point in a developmental trajectory that identifies actively dividing cells
- newly included, deeper analysis of the heat shock experiment

We believe we have fully addressed the reviewers' comments and suggestions. Please see our point-by-point response for details. Following the instructions of the editor, we did not consider the request to perform in situ hybridization experiments (reviewer 3) as these are beyond the scope of this study.

Reviewer #1:

Jean-Baptiste et al. present a single cell RNA-seq analysis of 3,000 root cell protoplasts as well as a comparison between a smaller number of single cell transcriptomes of heat-shocked and control root protoplasts. Analyses of these data in comparison with available transcriptomes from flow-sorted protoplasts enabled them to assign each cell to a specific type, and the use of pseudotime ordering allowed them to further examine the developmental trajectory of several cell types. This revealed known and previously unknown genes with cell type-specific expression, as well as the presumptive transcription factors that regulate many of these genes. The comparison between control and heat-shocked cells sought to determine whether the transcriptional response to stress varied across cell types. This analysis confirmed that the response is in large part uniform across cells, but some potentially interesting differences were also observed. Overall, this is an excellent piece of work that shows the successful application of this developing technology to plants and also provides interesting new biological insights. My only issue with the manuscript is in the low level of detail in some areas, which can be easily remedied.

Point 1. Both the introduction and discussion are very short and would benefit from expansion. For example, given that this approach is new to plant biology it would be useful to have more background on single-cell RNA-seq methods as well as some discussion of the advantages and limitations.

RESPONSE: We expanded the introduction and discussion significantly.

Page 2, Lines 56-68:

"While several examples of single cell RNA-seq have been carried out in Arabidopsis (Efroni et al., 2016, 2015; Brennecke et al., 2013), they were restricted to only a few cells or cell types. No whole organ single-cell RNA-seq has been attempted in any plant species. The Arabidopsis examples focused on root tips, finely dissecting the dynamics of regeneration or assaying technical noise across single cells in a single cell type. Thus, a need exists for larger scale technology that allows a more complete characterization of the dynamics of development across many cell types in an unbiased way. Such technology would increase our ability to assay cell types without reporter gene-enabled cell sorting, identify developmental trajectories, and provide a comparison of how different cell types respond to stresses or drugs. Several high-throughput methods have been described for sequencing of RNA at a high throughput of single cells. Most of these, including most droplet-based methods, rely on the 3' end capture of RNAs. However, unlike with bulk RNA-seq, the data from single cell methods can be sparse, such that genes with low expression can be more difficult to study."

Page 17, Lines 518-531:

"However, analyzing stele cells separately yielded 6 sub-clusters, which correspond to known vasculature cell types. Our approach to annotate these sub-clusters exemplifies the ad hoc nature of current single-cell genomics studies, which require all available sources of information to be exploited to interpret the genomic data. Neither Spearman rank correlations with sorted bulk RNA-seq data nor microarray expression data yielded obvious cluster identities. However, mean expression values of genes known to be expressed in vasculature cell types allowed us to assign the stele sub-clusters.

We identified hundreds of novel genes with cell-type-specific and tissue-type-specific expression, which may allow the generation of new marker lines for detailed genetic analyses. These genes, together with cluster-specific enriched transcription factor motifs and their corresponding transcription factors, are candidates for driving differentiation and cell-type identity."

Page 18, Lines 549-561:

"While several examples of single cell RNA-seq have been carried out in Arabidopsis (Efroni et al., 2016, 2015; Brennecke et al., 2013), they were restricted to only a few cells or cell types. No whole organ single-cell RNA-seq has been attempted in any plant species. The Arabidopsis examples focused on root tips, finely dissecting the dynamics of regeneration or assaying technical noise across single cells in a single cell type. Thus, a need exists for larger scale technology that allows a more complete characterization of the dynamics of development across many cell types in an unbiased way. Such technology would increase our ability to assay cell types without reporter gene-enabled cell sorting, identify developmental trajectories, and provide a comparison of how different cell types respond to stresses or drugs. Several high-throughput methods have been described for sequencing of RNA at a high throughput of single cells. Most of these, including most droplet-based methods, rely on the 3' end capture of RNAs. However, unlike with bulk RNA-seq, the data from single cell methods can be sparse, such that genes with low expression can be more difficult to study."

Point 2. In line 64 - what is the definition of a unique molecular identifier (UMI)?

RESPONSE: UMIs are random 10 base tags added to the cDNA molecules. We have added the following text to clarify the nature of UMIs on page 3, lines 82 and 83.

"UMIs here are 10 base random tags added to the cDNA molecules that allow us to differentiate unique cDNAs from PCR duplicates."

Point 3. In Figure 1 - what exactly is a Louvain component? A bit of explanation would be worthwhile here.

RESPONSE: Louvain components refer to clusters of cells with similar gene expression profiles derived with the Louvain method. We have added some additional text to address this comment and explain this method in more detail on page 4, lines 122-129:

"Louvain components were derived using the Louvain method for community detection (Blondel et al., 2008) which is implemented in Monocle 3. Unlike k-means clustering for which the user provides the desired number of clusters to partition a dataset, Louvain clustering optimizes modularity (i.e. the separation of clusters based on similarity within a cluster and among clusters), aiming for high density of cells within a cluster compared to sparse density for cells belonging to different clusters. The 11 clusters presented in Figure 1A optimized the modularity of the generated expression data and were not defined by us."

Point 4. In lines 182-185: the specific TF names are used in the text but the gene ID numbers are used in the corresponding Supplemental Figure 4, which makes cross referencing difficult.

RESPONSE: We apologize for making this challenging. We have corrected this oversight and now denote each gene with its systematic name and common name (if existing) in both text and figure legends.

Point 5. Regarding lines 227-228: Were the single root hair cell transcriptomes compared to whole tissue from the 13 developmental sections, or to the sorted COBL9 protoplasts from each section? It is not clear to me how the term "bulk" is being used in this context.

RESPONSE: We are using two different "bulk" data sets for comparisons, an earlier microarray-derived one (Brady et al., 2007) and a more recent RNA-seq data set (Li et al., 2016). Both data sets rely on sorted protoplasts from cell-type-specific reporter lines. We are also using a set of 530 cell-type-specific marker genes published by Cartwright et al., 2009 for additional analyses. In the revised manuscript, we have attempted to clarify in the main text which data set is used; we also annotated figures and figure legends accordingly.

For example on page 10, line 302-310, we now write:

"To dissect the developmental dynamics of individual clusters, we first focused on the well-defined root-hair cells, in which combined single-cell expression values highly correlated with those from bulk protoplasts sorted for expression of the COBL9 root-hair marker gene microarray data (Supplemental Table 1, Brady et al., 2007). To

annotate the unsupervised trajectory that Monocle 3 created for hair cells, we used the Spearman's rank test to compare expression in all cells to bulk expression data representing 13 different developmental stages in root tissues from all the available sorted cell types (Supplemental Figure 6) (Brady et al., 2007; Cartwright et al., 2009). Each cell was assigned the developmental stage and cell type most correlated with its expression values"

Point 6. In Figure 3, Panels C and D, are we really looking at the total RNA level (y-axis) or is this the number of expressed genes? If it is the former, then I don't understand how this was measured. Related to this (lines 241-244), I don't understand how transcription rate is being deduced from the total RNA amount, given that RNA abundance is a function of both synthesis and degradation. It is also unclear to me how RNA velocity analysis works (first mentioned in lines 252-254).

RESPONSE: Please note that these figures are Fig. 4D and E. These figures depict the median total RNA molecules for the number of genes indicated in each graph. In Fig. 4D, we show the median total number of RNA molecules for 10,201 genes (expressed in at least 5% of hair cells) across pseudotime. Unlike bulk RNA-seq, single-cell RNA-seq can determine the total number of RNA molecules measured per cell, allowing us to derive the median total RNA molecules across any cell type.

RNA velocity is a new measure of transcription rate; it is based on the ratio of spliced to unspliced RNA. The method assumes that increased levels of unspliced transcripts correlate with higher transcription rates. We have added some clarification to the main text on page 11, lines 335-346.

"To further explore this transcriptional dynamic, we calculated RNA velocity (La Manno et al., 2018), a measure of the transcriptional rate of each gene in each cell of the hair cell cluster. RNA velocity takes advantage of errors in priming during 3' end reverse transcription to determine the splicing rate per gene and cell. It compares nascent (unspliced) mRNA to mature (spliced) mRNA; an overall relative higher ratio of unspliced to spliced transcripts indicates that transcription is increasing. In our data, only ~4% of reads were informative for annotating splicing rates, a lower percentage than what has been used in mammalian cells for velocity analyses, and thus our results may be less reliable. Based on data for 996 genes, mean RNA velocity increased across pseudotime (Supplemental Figure 7B, p = 2.2 e-16 linear model, rho = 0.73). This increase in velocity was associated with the predicted changes in endoreduplication (Bhosale et al., 2018), especially between the 4N and 8N stages (Supplemental Figure 7C, Tukey's multiple comparison p-value = 0.0477)."

Point 7. In Supplemental Figure 9: What is the scale in panel C? Please provide more explanation for the analysis in panel D.

RESPONSE: In the revised manuscript, we have omitted Supplemental Figure 9C because its information overlaps with other figure panels. Similar information can be found in Supplemental Figure 12A, which highlights the expression of previously described heat shock genes.

We have expanded Supplemental Figure 9D (now Supplemental Figure 11A) in the revision and provide a more extensive explanation for the analysis conducted in the main text on page 14, lines 430-434.

"Upon heat shock, many cells, especially those with non-hair, phloem and columella as their highest rank, commonly showed as their second highest rank a different cell type instead of another developmental time point of the same cell type as observed in control cells (Supplemental Figure 11A)."

Point 8. Regarding Figure 5C, it is not clear to me how cell types were assigned, as this seems to contradict the earlier statement that such categorization was not possible due to the drastic changes in cell type-specific marker genes (lines 312-316). This section of the paper would benefit from more explanation.

RESPONSE: We appreciate the opportunity to further clarify this section of our manuscript. In our heat shock sample, we observed a global downregulation of gene expression accompanied with upregulation of heat shock genes (*e.g.* Figure 7B, D, E). This global down-regulation included the marker genes previously used to assign cell types (see Figure 7B, right volcano plot). To allow cell type identification in our heat shock sample, we used the mutual nearest neighbor batch correction algorithm (Haghverdi et al., 2018) to embed cells from untreated and treated conditions in a UMAP graph after correcting for differences in gene expression due to heat shock treatment. This approach allows for joint cell type assignment based on gene expression of control cells. The approach was performed as follows: First principal component analysis was performed on untreated and heat-shock treated cells. Then the PCA coordinates of untreated and treated cells were supplied to the mutual nearest neighbor algorithm.

Nearest neighbor identifies which cells in our heat shock sample are more similar to cells in our untreated control sample as defined by their entire transcriptome signature, not just a predefined set of marker genes. Then a correction is applied to align control and heat shock samples in a UMAP graph, where each cluster is now composed of cells from both conditions where the effect of heat shock has been subtracted (*i.e.* similar cells from the untreated and heat-shock treated cluster in similar areas of the UMAP embedding). Next, cell type assignment was made on the resulting clusters leveraging the cell-type marker gene expression of control cells that cluster with the "corrected" heat-shock treated cells. Importantly, as this correction is only used to create the UMAP graph and does not change the underlying expression matrix it allows for identification of heat-shock-specific gene expression programs at the cell-type level.

The section explaining this method has been clarified and can be found on page 14, lines 436-444:

"To enable such comparisons, we used a mutual nearest neighbor to embed cells conditioned on treatment in UMAP space (Haghverdi et al., 2018). The mutual nearest neighbor method was originally developed to account for batch effects by identifying the most similar cells between each batch and applying a correction to enable proper alignment of data sets. Here, we employ this technique to overcome the lack of marker expression in our heat-shock treated cells and match them to their untreated counterpart based on overall transcriptome similarity (Figure 7A). This procedure yielded corresponding clusters in control and heat-shocked cells, albeit with varying cell numbers for most (Supplemental Figure 11C, Supplemental Table 2)."

Reviewer #2:

The ms by Jean-Baptise et al. on "Dynamics of gene expression in single root cells of A. thaliana" details a high-throughput single-cell RNA-seq analysis of a well-studied organ. The ms can be considered a "proof-of-principle" study as it attempts a de novo reconstruction of a plant organ in a system that affords a good deal of corroborating data. That is a novel endeavor so it has value to the plant community. However, the paper has a good deal of analysis that is not always clear. Some of the conclusions seem based on vague or difficult to parse analysis and it is not always clear what conclusion the authors are trying to extract from the data. The lack of clarity extends to the claims of novelty as the authors make some claims about the novelty of single-cell RNA-seq analysis in plants yet do not cite any of the previous papers on single-cell RNA-seq in plants. If stated clearly, I do think there is novelty in the proof of concept for this scale of analysis, and the ms could represent an important technical step.

Point 1. The paper is largely about the technical advance that will enable the deconstruction of a plant organ transcriptome cell-by-cell. The authors chose perhaps the best-described organ in plants for their purposes, which is a fine choice for the sake of validation. However, given the choice of model, I find the validation superficial using nine genes with known expression patterns while there are many more available. There should be a wider and quantitative analysis of the agreement between known patterns and their clustering and cell-type classification routines.

RESPONSE: We apologize for not describing our methods of cell-type and tissue-type assignments more explicitly as this would have avoided this misunderstanding. We agree that using only nine genes for assignments and validations would be egregious. In fact, we take advantage of two different "bulk" data sets for assignments and validations, an earlier microarray-derived one (Brady et al., 2007) and a more recent RNA-seq data set (Li et al., 2016). Both data sets used sorted protoplasts from cell-type-specific reporter lines. The former is used in Figure 1B, Figure 4A, Figure 5A, Supplemental Figure 6, 8, 11 for cell-type assignments based on Spearman's rank correlations. The latter is used in Figure 1D, E, and Figure 4B for validations. In the revised manuscript, we also use the RNA-seq data set for cell-type assignments based on Pearson's correlations with expression profiles in each cell (Supplemental Figure 2). Moreover, we also exploit a set of 530 cell-type specific marker genes published by Cartwright et al., 2009 for additional analyses (e.g. Figure 1C, Supplemental Figure 3). All three methods together yielded the robust cell-type assignments we present in Figure 1A. In the revised manuscript, we have attempted to clarify in the main text which data set is used for each analysis; we also annotated figures and figure legends accordingly.

Point 2. I didn't understand what the authors were trying to say in the paragraph starting at line 169. What did the mutant analysis show? One would expect that targets would be affected in a cell-specific manner but this is not the analysis? Overall, it's not clear what the details in the next two paragraphs of the motif enrichment section is trying to

show. Primarily, again, there should be some quantitative test of whether the enrichment of TFs and their binding sites is indeed correlated. The validation right now seems anecdotal.

RESPONSE: We appreciate the opportunity to clarify this section of our manuscript. We identified enriched transcription factor motifs in several clusters. In plants, transcription factors often belong to large families. To explore which specific family member may drive a cell-type specific family motif enrichment, we used an admittedly imperfect approximation: expression of a specific transcription factor and family motif enrichment should occur in the same cluster.

We were similarly unclear in our description of the BZR/BEH mutants. We chose to focus on this family first because it is small and well-studied. We have recently provided evidence for partial functional redundancy within the BZR/BEH family, suggesting overlapping expression patterns. Indeed, we find these in root cells. In contrast, we describe several transcription factors for which expression overlaps with motif enrichments for their respective families.

Both of these sections have been substantially revised to facilitate understanding.

We now write on pages 7 and 8, lines 225-260:

“As transcription factors in A. thaliana often belong to large gene families without factor-specific motif information (Riechmann et al., 2000), it is challenging to deduce the identity of the specific transcription factor that drives cluster-specific transcription factor motif enrichment and expression. As an approximation, we examined transcription factors that were expressed in the cluster or tissue in which a significant enrichment of their motif was found, or in neighboring cell layers (some factors move between cells (Petricka et al., 2012)) (Supplemental Data Set 4). We focused first on the small BZR/BEH gene family whose motif was specifically enriched in cortex cells (cluster 10). Of the six genes (BEH1/AT3G50750, BEH2/AT4G36780, BEH3/AT4G18890, BEH4/AT1G78700, BES1/AT1G19350, and BZR1/AT1G75080) the single recessive beh4, bes1, and bzr1 mutants exhibit altered hypocotyl length (Lachowiec et al., 2018). Double mutant analysis suggests partial functional redundancy, which agrees with our observation of overlapping expression patterns for these genes across cell types (Supplemental Figure 5A, B). In contrast, neither beh1 and beh2 single mutants nor the respective double mutant show phenotypic defects (Lachowiec et al., 2018). However, BEH2 was the most highly expressed BZR/BEH family member across clusters and annotated root tissue and cell types (Supplemental Figure 5A, B). Although BEH4, the most ancient family member with the strongest phenotypic impact, showed cortex-specific expression, none of the BZR/BEH genes showed significance for cluster-specific expression, suggesting that combinations of family members, possibly as heterodimers, may result in the corresponding motif enrichment in cortex cells (Supplemental Figure 5A, B). In particular, BES1 and BZR expression was highly correlated, consistent with these genes being the most recent duplicates in the family (Supplemental Figure 5C) (Lachowiec et al., 2013; Lan and Pritchard, 2016).

In contrast to the BEH/BZR gene family, we found stronger cluster specificity for some TCP transcription factors. The TCP motif was strongly enriched in cortex (cluster 10), endodermis (cluster 1) and stele (cluster 7). Of the 24 TCP transcription factors, we detected expression for eight. Of these, TCP14 (AT3G47620) and TCP15 (AT1G69690) were expressed primarily in stele (clusters 7 and 4) although this cluster-specific expression was not statistically significant (Figure 2B, Supplemental Figure 5D, E, Supplemental Data Set 4). TCP14 and TCP15 are class I TCP factors thought to promote development. Acting together, TCP14 and TCP15 promote cell division in young internodes (Kieffer et al., 2011), seed germination (Resentini et al., 2015), cytokinin and auxin responses during gynoecium development (Lucero et al., 2015), and repression of endoreduplication (Peng et al., 2015). Both genes are expressed in stele in bulk tissue data (Brady et al., 2007; Winter et al., 2007), with TCP14 expression also observed in the vasculature by in situ hybridization (Tatematsu et al., 2008). TCP14 can affect gene expression in a non-cell-autonomous manner. ”

Point 3. The overall decrease in gene counts in the developmental trajectories for hair cells (Fig 3) but higher counts for cell type specific genes late in the trajectory was interesting in that it is a primary observation about differentiation that could not have been made with bulk sorts. The discussion seems to imply this was unique to hair cells but it's not clear where the authors do the same analysis in other cells types (cortex?). All cells are undergoing differentiation so specialization should be occurring across all cell types. More analysis of the developmental trends that could only be made with individual cell-type trajectories was critical, but these important points were left unclear.

RESPONSE: We agree that this analysis highlights the promise of single-cell genomics. We focused on hair cells

because they were prevalent in our data set and yielded the clearest developmental trajectory. As requested, we have now included trajectories for both cortex (Supplemental Figure 9A-C) and epidermis (Supplemental Figure 8F-H). We also describe these analyses in the revised manuscript on page 12, lines 359-362.

"Although we observed some decrease in total RNA expression and increased expression in cell-type specific genes for endodermis, we did not see a clear pattern of change in total RNA across cortex pseudotime (Supplemental Figures 8 & 9)."

Point 4. The heat shock analysis was also unclear. If many cell type specific genes were no longer regulated in a cell type specific fashion, how reliable was the nearest neighbor analysis in realigning these two different conditions? It seems to be implied in the discussion that this was a difficult problem but it is not detailed well in the results. E.g., the alignment of the two datasets looks almost perfect in Fig. 5A?

RESPONSE: We apologize for being unclear. A similar concern has been voiced by reviewer 1. See response to Reviewer #1, point 8.

Point 5. The authors' representation of the novel ground covered by the work is another aspect of the ms that is not clear. In the intro, they discuss bulk sampling in plants and then seem to imply that their novelty is single-cell RNA-seq in plants: "Single-cell RNA-seq analysis has been applied to heterogeneous samples of human, worm, virus ... Here we explore the potential of single-cell RNA-seq to capture the expression of known cell-type specific genes and to identify new ones." In discussion, "Despite its potential, single-cell RNA-seq has not heretofore been broadly applied to plants." But there have been at least three single-cell RNA-seq studies previously published on plants (Brennecke et al. 2015, Efroni et al. 2015, Efroni et al. 2016). Why aren't these cited?

RESPONSE: We thank the reviewer for pointing out this oversight. We have added text to the introduction; see response to Reviewer #1, comment 1.

Point 6. Overall, the authors are applying new techniques (i.e. droplet based commercial technologies that greatly increase the scale of single-cell RNA-seq) to plant cells. This allows them to profile an entire organ without any supervised selection of cells. But the corroboration of the data is not very deep given the amount of data they could have used. Of course they are relying on a system where the supervised collection has taken place already so it behooves them to be thorough on the issues of how successful this could be without the prior information, what novel types of information are gained by the single cell RNA-seq analysis at this scale over bulk sampling (e.g., were any "new" cell types identified, as per intro?), and how robust the data is compared to bulk samples of both cell types and developmental zones.

RESPONSE: We agree that we had the unique opportunity to rely on the extensive tools and data sets for the *A. thaliana* root. The correspondence of our data with this vast prior knowledge gives us confidence that single-cell RNA-seq will be a useful tool in plant genomics. We did not find new cell types, nor did we expect to find any in this well-studied organ with its simple anatomical features. The main novel findings of our manuscript are (i) the developmental trajectories within several cell types; (ii) the characterization of dividing cells within a trajectory branch point; and (iii) the observation of varying responses to a severe abiotic stress across cell types. With regard to the question of how our data compare to sorted RNA-seq and microarray data, please see Figure 1F, which compares cell annotations and proportions for sorted bulk data and single-cell data to microscopy.

<u>Reviewer #3:</u>

In this study, Jean-Baptiste and colleagues use single-cell RNA-seq to explore the transcriptional diversity of cell states in the root of Arabidopsis thaliana. They have used the droplet-based assay developed by 10x Genomics to generate single cell transcriptome profiles for 3,121 cells of the root.

The authors first identified the major known cell types of the root by correlating gene expression values in single cells with previously published bulk RNA-seq data from sorted cell-types. Once the cell types were identified by this approach, the authors undertook analyses that aim to define new transcriptional signatures of the different cell populations and the transcription factors that potentially drive them. They also ordered the cells of some lineages of the root in pseudotime to try to explore the spatiotemporal control of cell differentiation in the root. Finally, they took

advantage of this technology to identify cell-type-specific responses to heat stress, by analysis of another set of data from 2,085 cells in total.

Single-cell technologies, developed in the past few years, are opening up new ways of tackling fundamental biological questions by combining the comprehensive nature of genomics with the microscopic resolution that is required to describe complex multicellular organisms at single cell resolution. While these methods have been extensively applied to animal systems, very little has been done so far in plants. As the authors suggest in this study, single-cell RNA-seq will be crucial for gaining a much better understanding of the mechanisms at play during plant development and in response to their environment. However, the study as presented here contains a number of serious limitations that should be addressed by the authors, as outlined below.

Overall, the major weakness of the study is that the authors have used an extremely powerful technology but haven't really taken full advantage of the information it produces. Consequently, the new biological insights gained from this study are somewhat limited. Most of the analyses are largely used to confirm previously published results: the clustering defines fewer cell types than previously reported through reporter gene-enabled cell type labelling and sorting experiments; the transcription factor analyses focus on previously reported mutants; and the pseudotime trajectories are matched to previous data without exploring the unexpected complexity that appears to be present in the results. Further developing the heat stress component of the study, which seems to be the aspect that encompasses most of the biological novelty, could be a good approach to improve the manuscript and add significant new biological insights.

Point 1 - The process of disrupting the cell wall by protoplasting (prior to cell sorting or single-cell experiments) is known to affect gene transcription, and the effect of this upon the results and their interpretation must be considered. How was the effect of protoplasting on gene expression accounted for? What is the effect of removing genes affected by the protoplasting process from consideration in the analyses? Are there cell type specific protoplasting responses?

> RESPONSE: We thank the reviewer for this comment and agree that this deserved to be analyzed. According to Birnbaum et al. 2003, 346 genes change in expression in response to protoplasting, 76 of which were included in our 1500 ordering genes. Removing these genes had little effect on UMAP visualization, Louvain components and cell types. Therefore, these genes were kept in the subsequent analyses. We did, however, discuss this potential problem and its resolution in the manuscript on page 6, lines 172-178.
>
> "Protoplasting, the removal of the plant cell wall, alters the expression of 346 genes (Birnbaum et al., 2003); 76 of these genes were included in the 1500 genes with the highest variation in expression (Supplemental Data Set 1, Supplemental Figure 1B) that we used for clustering. Some of the 76 genes showed cell-type-specific expression. To exclude the possibility that the expression pattern of these genes produced artefactual clusters and cell-type annotations, we removed them from the analysis and re-clustered, which resulted in a similar UMAP visualization, with similar numbers of Louvain components and cell types."

Point 2. Despite being robust, droplet-based methods are not perfect: a certain proportion of the droplets generated will contain more than one single cell. Has an attempt been made to identify and remove such multiplets from the data? Can the number of multiplets be estimated?

> RESPONSE: We thank the reviewer for this recommendation. In the revision, we have applied a previously published method to estimate multiplets in our data set. We have added the results to the main text on pages 2 and 3, lines 99-108. We have also added a description to the methods on page 22, lines 654-660.
>
> "Because some of the UMAP clusters, specifically clusters 9 and 11, consisted of cells that had higher than average amounts of nuclear mRNA, we were concerned that these clusters consisted merely of cells that were doublets, i.e. two (or more) cells that received the same barcode and that resulted in a hybrid transcriptome. As cells were physically separated by digestion, it was possible that two cells remained partially attached. In order to identify potential doublets in our data, we performed a doublet analysis using Scrublet (Wolock et al., 2018), which uses barcode and UMI information to calculate the probability that a cell is a doublet. This analysis identified only 6 cells, of 3,021 cells analyzed, as doublets, spread across multiple UMAP clusters and multiple cell types (Supplemental Figure 1E). Overall, given the low number of doublets, we did not attempt to remove these cells."

"Estimating doublets

Single-Cell Remover of Doublets (Scrublet) was used to predict doublets in our scRNA-seq data (Available at: https://github.com/AllonKleinLab/scrublet). Using Python 3.5, Scrublet was ran using default settings as described by the example tutorial which is available as a python notebook (Available at: https://github.com/AllonKleinLab/scrublet/blob/master/examples/scrublet_basics.ipynb). The only significant change was that expected double rate was set to 0.1, in the tutorial it is 0.06."

Point 3. Usually protoplasts are maintained at room temperature after being generated, for sorting for instance. Here, the authors placed the cells on ice during the time the cells were counted and processed. Could this potentially have an effect on gene expression in both normal and heat stress conditions?

RESPONSE: We agree that this protocol step may affect our results. However, it is typically used in single-cell RNA-seq to arrest transcription. We see strong correspondence with bulk data sets generated with sorted protoplasts (e.g. Figure 1F); we also observed a robust heat shock response in heat-shock-treated cells that were placed on ice. Taken together, it appears that the placement on ice had the desired effect of arresting transcription rather than inducing a cold response.

Point 4. The first dataset presented is composed of a unique batch. However, as a control for the heat stress experiment, data was generated for 1,076 additional cells in control conditions. Could these be used to control for batch effect or to simply increase the number of samples for the downstream analyses?

RESPONSE: This is another insightful comment and suggestion. Indeed, we could have corrected for batch effects and boosted our cell numbers for control cells. However, combining these experiments requires down-sampling to align the number of sequence reads per cell, leading to a reduction of complexity across the entire experiment. Moreover, if we combined data sets, we would have added only 1,076 cells to our 3,000+ cells. The smaller second sample is unlikely to add to the rarer cell types, which were underrepresented in the large first sample. In our opinion, this increase in cell number did not justify the far more complex data transformation and hence more challenging interpretation.

Point 5. To assess the diversity of cell states in the data, the authors have used the popular Louvain algorithm to subdivide the cells into clusters. It is stated that this method yielded 11 different clusters. How was this number determined? Single-cell data can be typically subdivided into different numbers of clusters based on a resolution parameter that is defined by the user. What is the rationale behind defining the dataset in these 11 clusters (rather than a different number)?

RESPONSE: The number of clusters is not defined by the user in our analysis. In the revised manuscript, we clarify this point in the text on page X, lines Y-Z.

"Louvain components were derived using the Louvain method for community detection (Blondel et al., 2008) which is implemented in Monocle 3. Unlike k-means clustering for which the user provides the desired number of clusters to partition a dataset, Louvain clustering optimizes modularity (i.e. the separation of clusters based on similarity within a cluster and among clusters), aiming for high density of cells within a cluster compared to sparse density for cells belonging to different clusters. The 11 clusters presented in Figure 1A optimized the modularity of the generated expression data and were not defined by us."

Point 6. The clustering or cell assignment could be improved. The authors describe a group of cells that have transcriptional signatures from both the columella and non-hair cells and identify those cells as non-hair cells. Could these simply be lateral root cap cells, which may exhibit common transcriptional features between these two sorted populations?

RESPONSE: We thank the reviewer for this thoughtful comment. In the revised manuscript, we have explored the early non-hair cells further by comparing our data for this cluster with sorted RNA-seq data. This comparison yielded even fewer columella cells. Instead, we found that many of the cells in Louvain component 8 correlated best with *WER*-sorted RNA-seq profiles. *WER* marks expression in both early non-hair and lateral root cap cells, which is consistent with the reviewer's suggestion that these cells represent a mix of lateral root cap and early epidermis cells. In the revision, we discuss this analysis and our findings in the main text on page 5, lines 136-145.

"Although some cells were most highly correlated in expression with the cell type columella in Spearman's rank

tests and RNA-seq Pearson's correlation, these cells co-clustered with non-hair cells (Figure 1B, Supplemental Figure 2). This finding is consistent with bulk RNA-seq data of sorted cells (Li et al., 2016). Specifically, the PET11 (columella) -sorted bulk RNA-seq data are most similar to bulk RNA-seq data sorted for GL2 and WER (Li et al., 2016), both of which mark non-hair cells (Petricka et al., 2012). Therefore, these cells were grouped as early non-hair cells with other non-hair cells in Louvain component 8. As their expression values were best correlated with RNA-seq data for WER-sorted cells, they likely represent a mix of early non-hair and lateral root cap cells, which have very similar expression profiles (Supplemental Figure 2)."

Point 7. One cluster is identified as being the stele, which is composed of many different cell types. The authors state that this cluster contains both phloem and xylem cells. Attempts should be made to identify the different cell types within this cluster.

RESPONSE: In response to this request, we have re-clustered the stele cells and identified several distinct sub-clusters. These sub-clusters were annotated as xylem, phloem, xylem pole pericycle, phloem pole pericycle, and phloem companion cells. We also identified novel genes that are expressed specifically in these cell types. This analysis and its results are now described on page 9, lines 274-291. The results are visualized in Figure 3.

"Clustering stele cells identifies novel genes with cell-type specific expression in the vasculature

Our initial attempts to annotate and separate cell types within stele tissue with marker gene expression or Spearman's rank correlations failed. Instead, we separately clustered stele cells to reveal 6 sub-clusters upon UMAP visualization, with 5 sub-clusters containing more than 40 cells. Their annotation via Spearman's rank correlation with sorted bulk data was not successful; however, using well-established marker genes expression, we detected cluster-specific expression patterns (Figure 3A and B).

Cells closely related to xylem pole pericycle constituted the largest group of cells (205 cells); phloem pole pericycle cells were the second largest (84 cells). The high number of pericycle cells likely reflects our experimental procedure, as these cells reside on the exterior of the vascular bundle. Both phloem and xylem clusters showed similar numbers of cells (77 cells and 72 cells respectively); the phloem companion cells formed a distinct cluster. We observed the expected sub-cluster expression for several known genes and marker genes and identified novel genes with sub-cluster-specific expression (Figure 3C, D, Supplemental Data Set 1). Although there was some discrepancy, especially for the APL gene, which is expressed in both companion and phloem cells (Figure 3C), this is largely due to missing data."

Point 8. Early in the manuscript the authors use two different methods for classifying cells: either assigning an identity to each cell individually, or assigning an identity to all cells in each cluster. When looking for cell-type specific markers the authors state: "Using cell-type annotations rather than Louvain clusters, we identified an additional 125 novel genes with cell-type-specific expression" (p.5). What motivated this choice? Is there a large discrepancy between the two approaches? If so, should the clustering then be revised?

RESPONSE: We did this analysis for both Louvain components and for our cell-type-annotated clusters, finding novel genes with specific expression in both cases. In total, 317 specific genes were found in the Louvain component analysis (164 of which were novel root cell type marker genes) and 507 in the cell-type-annotated clusters (125 of which were novel). The overlap between the two analyses was 314. This can be found in Supplemental Data Set 4. The Louvain components, in a few cases, encompassed one tissue in two components, including endodermis, stele and non-hair/lateral root cap. We therefore looked at specificity genes for both cell-type annotation and Louvain component.

We modified the text on page 6 on lines 191-198:

"Second, to find novel marker genes, we identified genes with significantly different expression within and among Louvain component clusters by applying the Moran's I test implemented in Monocle 3. We found 317 genes with cluster-specific expression, 164 of which were novel, including at least one in each cluster (Figure 2A, Supplemental Data Set 4). Using cell-type annotations rather than Louvain clusters, we identified 510 genes with cell-type-specific expression, of which 317 overlapped with the Louvain component cluster-specific expression genes, as well as an additional 125 novel genes, some of which have been implicated in the development of a cell lineage in targeted molecular genetics studies. "

Point 9. Developmental stages: I was very surprised that the single-cell data generated in this study correlated best with bulk data from the elongation zone rather than the maturation zone. In contrast to what is mentioned in the text (p.4), the 2cm of each root that was collected in this study should mainly consist of the maturation/differentiation zone rather than the elongation zone. Similarly, the pseudotime trajectory presented in Figure 3 seems surprisingly enriched for early stages, given the length of the harvested roots. The authors should comment on this. Overall, are they really looking at the different stages of maturation (that should be relatively rare) in their data, or could they instead be looking at variation of transcriptional states that occurs in the maturation zone when performing pseudotime analyses?

RESPONSE: We also found this interesting, positing that the most parsimonious reason for the elongation zone higher enrichment is that younger cells are easier to protoplast. This would also explain the enrichment of younger stages in Figure 3A (now Figure 4A). We have addressed this in the text as follows on page 5, lines 154-157:

"This observation is surprising given the more mature developmental stage of the harvested roots (Supplemental Figure 1A), and likely reflects that younger cells are more easily digested during protoplasting and contribute in greater numbers to the gene expression data."

Point 10. Pseudotime: The study presents a detailed pseudotime ordering of cells for the cortex, endodermis and hair cells independently. Based on correlation with previously reported bulk data from different sections of the root, the ordering seems indeed to recapitulate (to some extent) the differentiation process happening from the stem cell niche to the maturation zone. However, what is striking in all these trajectories, is that their topologies seem a lot more complex than expected. In the text, the authors completely ignore that their method has yielded trajectories with multiple branches (e.g. Figure 4), when one would expect a simple linear progression.

What is the biological significance of these branches? An analysis should be undertaken to identify specific markers for each of them.

RESPONSE: See point 12 below.

Point 11. An independent experimental approach such as in situ hybridization should be used to visualize the cells corresponding to these potentially interesting transcriptional states in vivo to determine what they could be, and to confirm that they really exist within an intact (i.e. not protoplasted) root.

RESPONSE: While we agree that this is a great idea for an additional new experimental direction, we believe it to be outside of the scope of the current research.

Point 12. Since the branching of the trajectories seems to be ignored, could it be that the genes that are identified as transiently expressed during maturation simply correspond to the different branches of the trajectory? Biologically, that would be something completely different.

RESPONSE: We did not force branchless trajectories in this analysis, which has subsequently let us determine whether there are biological processes enriched in these branches. The Figure 4 (now Figure 5) small branches in cortex are too short and the cloud of cells is too dispersed to identify cells that would be specific to these branches. We also looked at branches in Louvain component 9 (hair) and 8 (early non hair/ lateral root); while we found no significant biological processes in the hair branch, we did find interesting biology in the component 8 branch. We have created a subsequent figure and have included the following in the text both in the results and discussion.

Results changes can be found on pages 12 and 13 on lines 380-401:

"Branch points in developmental trajectories mark developmental decisions

Although a developmental trajectory that reflects the differentiation from early to late cells within a cell type should be branchless, we did observe some branch points, for example in Louvain component 8, affording us the opportunity to explore their biological relevance. As discussed, Louvain component 8 contains early non-hair cells and likely some lateral root cap cells. To further explore the cells within the branch, we performed a principal graph test, comparing their expression profiles to those of cells elsewhere in the cluster (Figure 6A). We found that cells within the branch were significantly enriched for expression of genes involved in cell plate formation, cytokinesis and cell cycle. We explored this enrichment for cell cycle annotations by comparing expression of previously identified core cell cycle genes (Gutierrez, 2009) in cells within the branch to cells in the rest of the cluster, finding

many core cell cycle genes, in particular many G2 genes, to be specifically expressed in branch cells (Figure 6B). Among these genes were several of the cyclin-dependent kinase B family members that direct the G2 to M transition. Two cyclin-dependent kinase subunits (CKS1 and CKS2), thought to interact with several CDK family members, were also specifically expressed in branch cells (Vandepoele et al., 2002). Other branch-cell-specific genes included AUR1 and AUR2, both involved in lateral root formation and cell plate formation (Figure 6C, Van Damme et al., 2011). Louvain component 9 also showed a strong, but short branching point. We did not find any biological processes enriched in genes expressed specifically in this short branch; however, one gene whose expression is known to be affected by protoplasting was specifically expressed in these cells, perhaps reflecting that cells within this branch were more stressed by our experimental procedure (data not shown)."

Discussion changes can be found on page 18 on lines 550-562:

"By allowing trajectories with side branches, we discovered that branch points can mark developmental decisions. In Louvain component 8, the small but distinct cell-cycle enriched branch may mark lateral root primordia cells differentiating into epidermal cells or epidermal/lateral root precursor cells. Cells within this branch express many cell cycle genes, among them members of the CDKB family that govern the G2 to M transition. Moreover, these cells specifically express the AUR1 and AUR2 genes, which function in cell plate formation; plants with mutations in these genes lack lateral roots (Van Damme et al., 2011). Although expression of cell cycle genes may persist in non-dividing cells because of their roles in endoreduplication, AUR1 and AUR2 expression (and cell plate formation) should not persist, consistent with our speculation that the cells within this branch are actively dividing cells in the G2 to M transition (Gutierrez, 2009). These side branch cells could be cells that were lateral root cap/ epidermis precursor cells, and the trajectory may the cell fate decision being performed for epidermis or lateral root cap. "

Point 13. The Arabidopsis cistrome study (O'Malley et al.) has demonstrated that transcription factor families rarely have a unique motif that they bind to. Which method was used to link transcription factor candidates to specific motifs?

RESPONSE: We used the cistrom study motifs for our analysis, keeping all enrichment at the family level. From there, we were interested in seeing if individual members of those families had interesting expression patterns over pseudotime, which is displayed in Supplemental Figure 10. We make no attempt at separating the motifs of family members, and simply use the motifs published in O'Malley et al.

Point 14. Care should be taken to not over interpret the data at times: "We asked whether we could determine which transcription factors govern the cluster-specific expression patterns" (p.7). Without additional experiments to demonstrate functionality, such conclusions cannot be made from the current data.

RESPONSE: We agree that this language was too strong, given that we have no proof for the role of these factors based only on expression pattern. We have changed the text thusly on page 7, lines 217 and 218:

"We asked whether we could determine which transcription factors could possibly contribute to the cluster-specific expression patterns."

Point 15. In the last part of the manuscript, the authors describe the expression patterns of the genes that are affected by heat stress and classify them into different cell clusters. It is a shame that this part of the study isn't further explored. There seems to be a lot of heterogeneity within the gene clusters, with cell-type specificities. A comparison between the two conditions per cell type or per cluster could be undertaken in order to attempt to better assess the subtlety of changes happening during this process.

RESPONSE: We thank the reviewer for this comment and in response have provided Supplemental Data set 5 of differential gene expression as a function of heat shock and cluster assignment to serve as an additional resource for readers. We further discuss these results in response to the reviewer comment below.

Point 16. There are far fewer cells in the control/treatment than in the initial dataset of 3k cells. Could there be some false-positives in the differential expression analysis that would be due simply to the process of subsampling a complex and diverse population of cells?

RESPONSE: We appreciate the reviewer comment and now clarify the nature of the heatmaps of gene expression across cells presented in Figure 5 (now Figure 7) and Supplementary Figure 9 (now Supplementary Figure 11). The approach outlined in our original submission sought to assign cell type identity to previously observed changes in

bulk sequencing assays, specifically, gene expression changes in response to heatshock from bulk RNA-seq analysis and gene expression changes observed for genes that reside in regulatory regions with known changes in chromatin accessibility upon heatshock treatment from bulk DNAse I accessibility (Alexandre et al., 2018; Sullivan et al., 2014).

However, in response to the reviewer comment above we now include Supplemental Data Set 6 of differential gene expression as a function of heatshock and cluster assignment. For this new analysis, we have taken into account the reviewer's concern regarding cell number and power to identify differential gene expression. We agree that the smaller number of cells in our heatshock experiment decrease our power to detect differential gene expression. With this in mind our approach to differential gene expression analysis does not include sub-setting cells by UMAP cluster. Instead we chose to identify differentially expressed genes (DEGs) across all cells using a full model of "y ~ Treatment*UMAP cluster" and a reduced model of "y ~ UMAP cluster". This full model should be understood to be "y ~ Treatment + UMAP cluster + Treatment:UMAP cluster" where our reduced model eliminates additive effects to arrive at DEGs altered due to treatment within UMAP clusters (which we assume to be individual cell types and/or cell states). Testing across all cells increases our power compared to DEG testing within individual UMAP clusters. Moreover, we expect that any loss of power associated with decreased cell numbers will have a larger adverse effect on our ability to call true DEGs. Additionally, as is standard for DEG analysis, we incorporate multiple hypothesis testing using the Benjamini-Hochberg method to control our false discovery rate. Lastly, as we identify a similar number of clusters as compared to initial scRNA-seq experiments, we are confident that our data set is large enough to detect cell specific differences. However, we cannot exclude the possibility that heatshock treatment leads to some cells mapping to incorrect clusters, which could indeed increase our false-positive rate.

Point 17. Figure 1F, regarding the relative representation of root cell types from these single-cell analyses and estimates from microscopy doesn't make much sense. The authors show some numbers that match for some cell types, but it doesn't match for a lot of them. The stele, for instance, should correspond to maybe half of the cells present in the root, which is not the case here. Protoplasting the cells will induce a bias in cell type proportion that doesn't necessarily compromise the study.

RESPONSE: This comparison was to highlight the fact that we captured some cell types better than others, likely due to the digestion process. We did not pre-process these roots at all so capturing the innermost vasculature the least efficiently makes sense. If this remains a major point of contention, it can be removed.

Point 18. The authors should be careful about the conclusions they draw from correlations. For instance, the fact that BEH2 has the same expression pattern across cells as BES1 doesn't make them functionally equivalent.

RESPONSE: We agree that interpretation is difficult in these cases and is a major reason that we do not dive much further into this topic amongst larger and more diverse transcription factor families (like the ARFs, for example). We have also softened the language based on this suggestion; please see the changes outlined in reviewer 1 comments.

Point 19. In the pseudotime figures, when using a color code for highest correlated developmental point, it might be better to use a description rather than numbers (e.g. "meristem", "elongation zone", etc). Another alternative would be to use a diagram showing where those different zones are located in the root.

RESPONSE: When we first apply this analysis and introduce this concept in Figure 3A (now Figure 4A), we have included a small graphic showing approximately where each section corresponds to on the root.

Point 20. In supplementary figure 10, the color scale is inverted compared to the other heatmaps in the manuscript (up to that point, yellow indicated the highest expression value), which is confusing for the reader.

RESPONSE: This was due to our error when manually adding the scale numbers. We have corrected this.

TPC2018-RA-00785R1 2nd Editorial decision – *acceptance pending* March 17, 2019

As we discussed in earlier correspondence, we only received one review of your revised manuscript. The second reviewer who agreed to assess your manuscript has not yet submitted a review, despite four reminders. Therefore to avoid further delay, we have decided to go ahead with the advice of the first reviewer and our reading of the

manuscript. We are pleased therefore to inform you that your paper entitled "Dynamics of gene expression in single root cells of A. thaliana" has been accepted for publication in The Plant Cell, pending a final minor editorial review by journal staff. At this stage, your manuscript will be evaluated by a Science Editor with respect to scientific content presentation, compliance with journal policies, and presentation for a broad readership. The Plant Cell has appointed several Ph.D. Plant Scientists to serve as Science Editors in this capacity, and you will receive further information on this process very shortly.

The small issues raised by the reviewer can be dealt with at the proof or science editorial stage.

Reviewer #1 (Comments for the Author):

I am satisfied with the authors' responses to each of the points I raised. Additional analyses that are now included make this paper even more interesting biologically than the original version. Before publication there are a few points that should still be clarified:

1. Line 99: Nuclear vs total RNA - do they mean unspliced vs spliced?

2. Regarding RNA velocity, on line 337, what does 3' end mispriming of cDNA have to do with this measurement?

3. Line 545, regarding transcription rates determined by RNA velocity - this apparent increase in velocity for endoreduplicated cells could alternatively be a manifestation of slower splicing due to limiting spliceosomal components.

Final acceptance from Science Editor	March 26, 2019
